# Novel Discorhabdin Derivatives from Antarctic Sponges of the Genus *Latrunculia*: Expanding the Chemical Diversity of Polar Marine Natural Products

**DOI:** 10.3390/md23100401

**Published:** 2025-10-15

**Authors:** Sam Afoullouss, Stine S. H. Olsen, Sydney Morrow, Ezequiel Cruz Rosa, Kaley Geu, Nerida G. Wilson, Bill J. Baker

**Affiliations:** 1 Department of Chemistry, University of South Florida, 4202 E. Fowler Avenue, CHE205, Tampa, FL 33620, USA; samafoullouss@usf.edu (S.A.); olsens@usf.edu (S.S.H.O.); kalyn12@usf.edu (S.M.); ecruzrosa@usf.edu (E.C.R.); geuk@usf.edu (K.G.); 2 School of Biological Sciences, The University of Western Australia, 35 Stirling Highway, Crawley, WA 6009, Australia; nerida.wilson@uwa.edu.au; 3 Collections & Research, Western Australian Museum, 49 Kew Street, Welshpool, WA 6106, Australia

**Keywords:** Discorhabdin, DFT, ECD, Antarctic, sponge

## Abstract

In this study, three Antarctic sponges of the genus *Latrunculia* were investigated, leading to the isolation of five unreported pyrroloiminoquinone alkaloids along with the known metabolite (+)-debromodiscorhabdin A (**3**). Three of the new metabolites were brominated, while the other two were found to have a C-5/C-8 sulfur bridge and a C-2/N-18 bridge. Three of the metabolites were shown to have a phenyl ketone substituent on C-14, not previously reported for discorhabdin derivatives. The cytotoxicity against the A549 cell lines was studied and compounds **1**–**4** showed activity of 4.3, 1.8, 1.0, and 23.9 µM, respectively, while no inhibition was found for **5** and **6**.

## 1. Introduction

Sponges of the genus *Latrunculia* (family: Latrunculiidae; class: Demospongiae) are known for producing cytotoxic pyrroloiminoquinone alkaloids known as discorhabdins, a name derived from the unusual discorhabd spicules that characterize the sponge family [1,2]. The sponges are commonly found in cold habitats, mainly Antarctica and the North Pacific [3]. They are known to produce a significant variety of secondary metabolites, but mainly the highly pigmented discorhabdins, many of which have shown strong activity against various cancer cell lines [4]. Discorhabdins described to date consist of a pentacyclic backbone of 18 carbons and 3 nitrogen atoms with a C-6 spiro-center and a C-11 ketone, or di- and trimers thereof [5,6]. The backbone displays various substitution including mono- and dibromination and a C-5/C-8 sulfur bridge [5].

**Figure 1:**
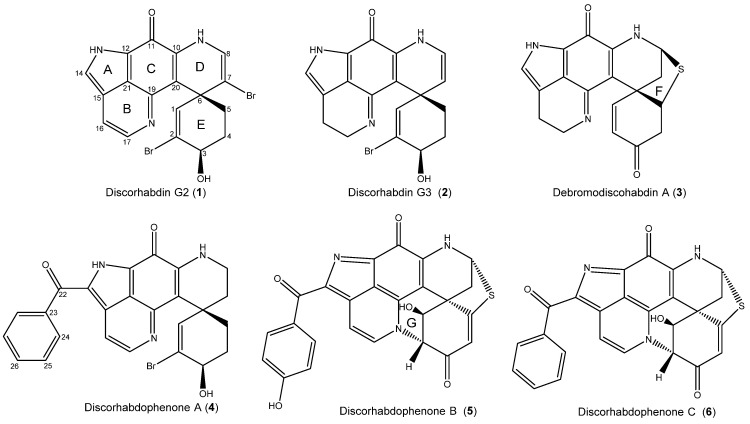
Alkaloids isolated from Antarctic sponges *Latrunculia* cf. *biformis* and *Latrunculia* sp.

The research discussed herein involves the investigation of three specimens of the genus *Latrunculia* sp. collected in Antarctica at two different locations. During a circumpolar cruise of Antarctica, led by the Swiss Polar Institute in 2016, two specimens of *Latrunculia* cf. *biformis* were obtained by trawl from the Kerguelen Plateau. A third, as yet unidentified, specimen of *Latrunculia* sp. was collected by SCUBA from the Palmer Archipelago in 2018. The metabolites were prioritized based on an MS-guided fractionation by pursuing fractions with a 1:1 or 1:2:1 isotopic pattern, indicative of mono- and dibrominated metabolites, combined with targeting aromatic ^1^H NMR signals. Five new metabolites with the discorhabdin backbone, illustrated in Figure 1, were isolated and characterized and one known metabolite, (+)-debromodiscorhabdin A (**3**), was identified through a comparison of its spectroscopic data to that previously published [7]. The metabolites were characterized through 1D/2D Nuclear Magnetic Resonance (NMR) Spectroscopy data, high-resolution mass spectrometry (HR-MS), and comparison of experimental and calculated chemical shifts, using DP4+ and Sorted Training Set (STS) comparison tools, and Electronic Circular Dichroism (ECD) spectra.

## 2. Results and Discussion

### 2.1. Structure Elucidation

Discorhabdin G2 (7-bromo-3-dihydro-16,17-dehydrodiscorhabdin G, **1**) was obtained from the 50% MeOH/H_2_O eluent from the *Latrunculia* cf. *biformis* specimen designated ACE16-49 (WAMZ44145). It was isolated as a deep-red solid with the pseudo-molecular ion peak at *m*/*z* 463.9450 ([M + H]^+^, HRESIMS) and a 1:2:1 isotopic pattern, indicative of two bromine atoms in the molecule. The ^1^H and ^13^C NMR data (Table 1) corroborated the molecular formula as C_18_H_13_Br_2_N_3_O_2_. The 18 carbon and 3 nitrogen atoms indicated that the compound had the backbone of the discorhabdins. The NMR data suggested that **1** had five olefinic protons; ten quaternary carbons, with nine of these being deshielded; one oxymethine; and two methylene groups.

**Figure 2:**
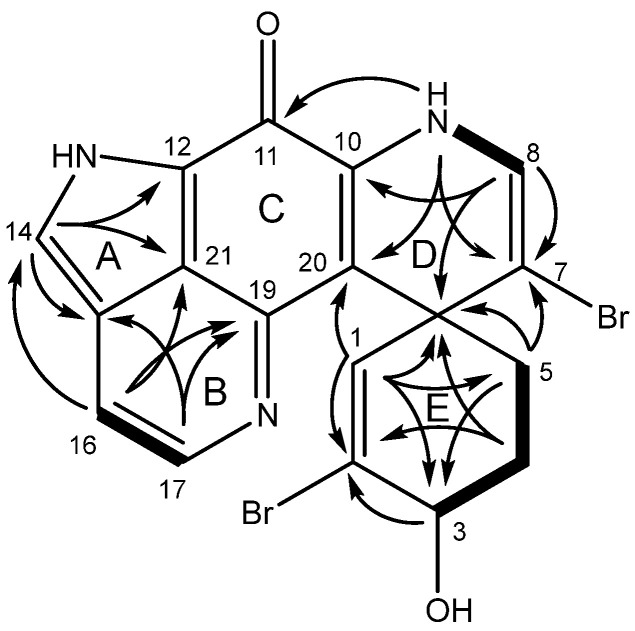
COSY (bold) and HMBC (arrow) correlations of **1**.

The structure was elucidated starting from homonuclear correlation spectroscopy (COSY) and Heteronuclear Multiple Bond Correlation (HMBC) correlations, as illustrated in Figure 2. Key correlations include COSY correlation between the oxymethine H-3 (δ_H_ 4.42) and H_2_-4 (δ_H_ 2.18/2.04) and further to H_2_-5 (δ_H_ 2.70/2.11). HMBC correlations were displayed from H-3 to the olefinic proton C-1 (δ_C_ 134.9) and quaternary carbon C-2 (δ_C_ 129.2). H-1 (δ_H_ 6.15) had HMBC correlations to C-2, C-3 (δ_C_ 67.3), C-5 (δ_C_ 36.9), C-6 (δ_C_ 45.9), C-7 (δ_C_ 104.0), and C-20 (δ_C_ 111.7). H_2_-4 and H_2_-5 showed HMBC correlations to C-3 and C-6, while H_2_-5 also showed correlations to C-7 and C-20. The high-field quaternary carbon C-6 was indicative of the spiro-center between rings D and E of the discorhabdin backbone, with C-3 often displaying an oxymethine. The H-8 (δ_H_ 6.60) olefinic proton had COSY correlation to the H-9 (δ_H_ 8.72) exchangeable proton and HMBC correlations to quaternary carbons C-6, C-7, and C-10 (δ_C_ 137.9), establishing the Δ^7^-olefin. Further HMBC correlations between H-9 and C-20 completed the assignment of the D and E rings of a discorhabdin skeleton, and another correlation to C-11 (δ_C_ 164.1) extended that ring system to the iminoquinone ketone.

The olefinic protons H-16 (δ_H_ 7.54) and H-17 (δ_H_ 8.31) showed COSY correlation to each other, while H-16 showed HMBC correlations to C-14 (δ_C_ 128.6), C-17 (δ_C_ 141.6), C-19 (δ_C_ 147.3), and C-21 (δ_C_ 119.5) and H-17 showed HMBC correlations to C-15 (δ_C_ 124.6), C-16 (δ_C_ 113.7), C-19, and C-21. This suggested that the olefin was on ring B. The remaining olefinic proton (H-14, δ_H_ 8.24) had HMBC correlations to quaternary carbons C-11 (δ_C_ 164.1), C-12 (δ_C_ 118.5), C-15, and C-21. Correlation from H-9 and H-14 on opposite sides of the molecule to C-11, indicated this deshielded quaternary carbon to be the carbonyl displayed in the discorhabdin backbone on ring C. The two bromines indicated by the HRESIMS were placed on the two open valences C-2 and C-7, completing the planar structure of **1**.

Discorhabdin G3 (3-dihydrodiscorhabdin G, **2**) was obtained from the same extract as **1** and was isolated as a green solid with the molecular formula C_18_H_16_BrN_3_O_2_, corroborated by the *m*/*z* of 386.0505 (HRESIMS, [M + H]^+^) and its ^1^H and ^13^C NMR data. The molecular formula indicated **2** to have three extra protons and one fewer bromine compared to **1**, while its ^1^H NMR spectrum suggested **2** to have two of the olefinic protons replaced by methylene groups. The 2D NMR data (Table S2) suggested the two compounds to display significant similarities, with the only differences being a lack of bromine at C-7 and reduction in the Δ^16^-olefin. The missing bromine was replaced by a new methine at δ_H_ 5.24 (H-7) with COSY correlation to H-8 (δ_H_ 6.28). The reduction in the Δ^16^-olefin was established by COSY correlation between the methylene protons H_2_-16 (δ_H_ 2.87) and H_2_-17 (δ_H_ 3.91) and HMBC correlations to C-14 (δ_C_ 126.8), C-15 (δ_C_ 119.4), C-17 (δ_C_ 44.8), C-21 (δ_C_ 122.4), and C-16 (δ_C_ 17.9) and C-19 (δ_C_ 157.2), respectively.

The 1D and 2D NMR data for **2** had significant similarities to the published data for discorhabdin G, published in 1995 by Yang et al. from *Latrunculia apicalis,* collected from the vicinity of McMurdo station in Antarctica [8]. Discorhabdin G was published with a molecular formula of C_18_H_15_BrN_3_O_2_, suggesting the loss of two protons compared to **2**. This matched the suggested structure elucidation with the C-3 ketone for discorhabdin G undergoing a reduction to the C-3 hydroxyl found for **2**.

A second specimen of *Latrunculia* cf. *biformis* from the 2016 sampling campaign, designated ACE16-51 (WAMZ144147), also produced two pyrroloiminoquinone metabolites, one of which was the known *Latrunculia*-derived metabolite debromodiscorhabdin A (**3**) based on comparison of NMR (Table S3) and mass spectral data to published values. Discorhabdophenone A (**4**) was isolated as a deep-red solid with a formula of C_25_H_20_BrN_3_O_3_, corroborated by ^1^H and ^13^C NMR data (Table 2 and Table S4) and the *m*/*z* of 490.0773 (HRESIMS, [M + H]^+^). The formula indicated that the structure had 7 additional carbons, compared to **1**–**3**, suggesting a significant difference from the discorhabdin backbone. The primary difference in the ^1^H NMR spectra was the presence of two 2H aromatic protons at δ_H_ 8.37 (d, H_2_-24) and δ_H_ 7.56 (t, H_2_-25), suggesting that the structure had a phenyl ring. This was confirmed using 2D NMR data that showed COSY correlation between the two, while H-25 had further COSY correlation to a 1H olefinic proton signal at δ_H_ 7.63 (H-26). Phenyl rings are unknown from previously described discorhabdin derivatives. Furthermore, H-25 had HMBC correlation to C-23 (δ_C_ 138.9) and H-24 had HMBC correlation to the deshielded C-22 (δ_C_ 189.1), suggesting a benzoyl substituent.

Other than that benzoyl group, the NMR data suggested a discorhabdin backbone with the presence of the C-6 spiro-center (δ_C_ 38.7), the C-3 oxymethine (δ_C_ 68.0) (C-3), and the C-11 deshielded quaternary carbon (δ_C_ 169.2). The 1D and 2D NMR data showed the backbone of **4** to have significant similarities to **1** with the only changes being two methylene groups in the place of the Δ^7^-olefin, the C-7 bromine, and the loss of the proton previously seen on C-14. To complete the planar structure of **4**, the benzoyl substituent provides a ketone bridge between the phenyl ring and the open valence on C-14. While no correlations from H-16 to C-22 or H-24 to C-14 were present in multiple HMBC or LR-HMBC experiments, the open valence on C-14 offers the only plausible location for the benzoyl group.

The *Latrunculia* sp. specimen from Palmer Station yielded two pyrroloiminoquinones with significant similarities to each other. One, discorhabdophenone B (**5**) displayed two 2H olefin doublets (H_2_-24, δ_H_ 8.42; H_2_-25, δ_H_ 6.90) compared to the second, discorhabdophenone C (**6**), with one olefinic doublet (H_2_-24, δ_H_ 8.35) and two olefinic triplets (H_2_-25, δ_H_ 7.55; H-26, δ_H_ 7.63). These shifts suggested that **5** had a *p*-disubstituted phenyl ring and that **6** had a mono-substituted phenyl ring, as found in **4**.

**Figure 3:**
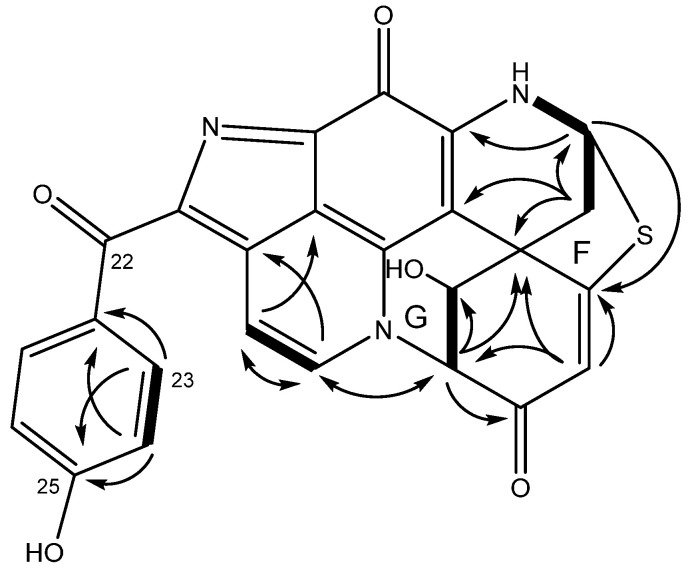
COSY (bold) and HMBC (arrow) correlations of **5** establishing new rings F and G.

Discorhabdophenone B (**5**) was isolated with a *m*/*z* of 470.0810 (HRESIMS, [M + H]^+^) and a molecular formula of C_25_H_15_N_3_O_4_S, corroborated by the ^1^H and ^13^C NMR data (Table 2). Structure elucidation was completed by COSY and HMBC correlations (Figure 3) and comparison with known discorhabdin derivatives. The compound was identified as a discorhabdin due to the C-6 spiro-center (δ_H_ 47.5) and the C-11 carbonyl (δ_C_ 168.7). The ^1^H NMR data indicated that **5** had a *p*-substituted phenyl ring, two additional aromatic protons, four deshielded methines, and one methylene.

Discorhabdophenone B (**5**) differed from the previously presented discorhabdins herein due to the presence of a sulphur atom in the molecular formula; however, a C-5/C-8 sulphur bridge is a common feature among discorhabdin derivatives. Additional differences were the presence of three deshielded methines and the absence of the bromine atom, identified by the lack of a 1:1 isotopic pattern in the mass spectrum (Figure S36), and the presence of two extra deshielded quaternary carbons. The two deshielded methines H-1 (δ_H_ 4.84) and H-2 (δ_H_ 5.02) had COSY correlation to each other, with H-1 being identified as an oxymethine due to COSY correlation between the exchangeable δ_H_ 6.75 (OH-1) and H-1. H-2 displayed HMBC correlations to deshielded C-1 (δ_C_ 67.2), C-3 (δ_C_ 184.5), C-6 (δ_C_ 47.5), and C-17 (δ_C_ 131.5). H-4 (δ_H_ 5.96) had HMBC correlations to C-2 (δ_C_ 65.7), C-5 (δ_C_ 171.3), and C-6, identifying ring E, as shown in Figure 3, with a ketone on C-3, Δ^4^ olefin, and an open valence on C-2 and C-5.

**Figure 4:**
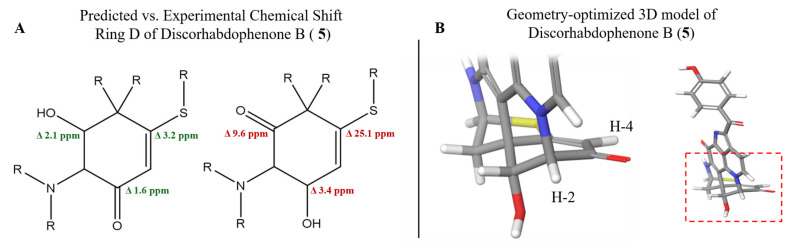
(**A**) Predicted vs. experimental chemical shifts in possible regioisomers of discorhabdophenone B (**5**) measured in delta ppm. (**B**) 3D model of geometry-optimized conformer of discorhabdophenone B (**5**) showing H-2 and H-4 are in the same plane of ring D.

The lack of a clear HMBC correlation from H_2_-7 to C-1 left a level of uncertainty regarding the position of the carbonyl and hydroxy group on ring D. The chemical shift in the carbonyl suggested that it was part of a conjugated system, positioning it on C-3. Conversely, TOCSY correlations displayed evidence of a spin-coupled system between H-1/H-2/H-4, supporting the assignment of the hydroxy group to C-3. To determine the location of the carbonyl, DFT-based chemical shift predictions for the regioisomers of **5** were compared to the experimental chemical shift. The predicted chemical shifts in both thiol–ene and carbonyl carbons were in significantly better agreement with the experimental chemical shifts with the hydroxy group on C-1 and the carbonyl on C-3 (Figure 4A). Examination of the geometry-optimized conformer of **5** showed that H-2 and H-4 occupy the same plane (<10°). This is consistent with W-coupling between H-2/H-4, explaining the observed H-1/H-2/H-4 TOCSY spin system, while no direct COSY correlation from the hydroxyl (H-1) to H-4 was observed (Figure 4B).

The third deshielded methine H-8 (δ_H_ 5.70) displayed COSY correlations with methylene H_2_-7 (δ_H_ 2.86/2.61) and the exchangeable H-9 (δ_H_ 9.30). Furthermore, H_2_-7 showed HMBC correlations to C-6, C-8 (δ_C_ 63.4), and C-20, placing these in ring D. The open valences on C-5 and C-8 suggested the sulphur bridge previously mentioned. This was corroborated by HMBC correlations from H-8 to C-5. The two olefinic protons δ_H_ 7.94 (H-16) and δ_H_ 8.05 (H-17) were left to assign, with COSY correlations shown between the two and HMBC correlations from H-16 to C-19 (δ_C_ 146.0). H-17 had HMBC correlation to C-2, suggesting an N-18 to C-2 heterocycle, as reported for previous discorhabdin derivatives. Lastly, the previously established benzoyl group was connected to the discorhabdin backbone at the only remaining open valence, C-14, as observed in **4**.

Discorhabdophenone C (**6**) was isolated with the *m*/*z* of 454.0861 (HRESIMS, [M + H]^+^). Corroborating ^1^H and ^13^C NMR data (Table 2), **6** was found with the molecular formula of C_25_H_15_N_3_O_4_S, which is one oxygen atom fewer than found in **5**. The 1D and 2D NMR data indicated a significant difference for C-26 (δ_C_ 161.5 for **5** and 132.1 for **6**), suggesting that **6** lacked the phenol function and congruent with the ^1^H NMR analysis of the two that was described above. Unlike the reported discorhabdins which contain the C-2 to N-18 bridge, compounds **5** and **6** were not isolated in a protonated state. This was determined based on the number of exchangeable protons visible in ^1^H NMR spectra, and the presence of sodium adducts [M + Na]^+^ (**5**: calculated *m*/*z*: 492.0635, observed *m*/*z*: 492.0613, Δ 4.6 ppm; **6**: calculated *m*/*z*: 476.0681, observed *m*/*z*: 476.0679, Δ 0.4 ppm) in HRESIMS data (Figures S37 and S49) [9,10,11].

### 2.2. Stereochemical Analysis

#### 2.2.1. Relative Stereochemistry

**Figure 5:**
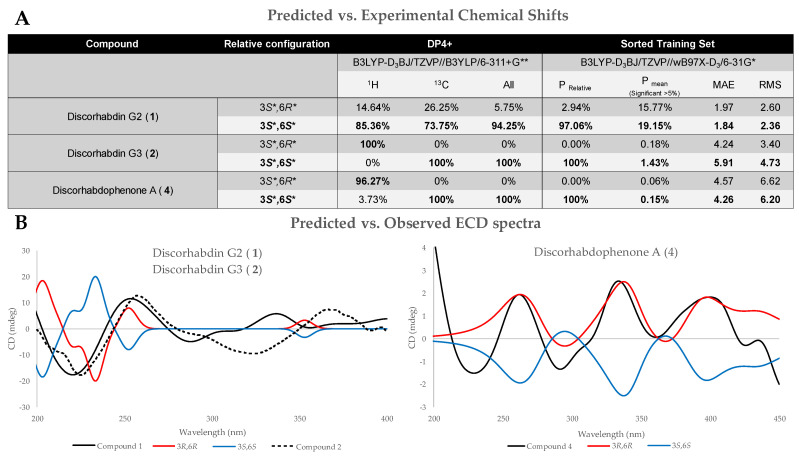
(**A**) DP4+ and STS probabilities from comparing experimental and DFT-predicted chemical shift in relative configurations of compounds **1**, **2**, and **4**, with results indicating 3S*,6S* configuration. (**B**) Experimental vs. observed ECD spectra of **1**, **2**, and **4**. Spectra were predicted and measured in methanol.

The stereochemistry of the new discorhabdin derivatives reported herein (**1**, **2** and **4–6**) was established based on coupling constants and chemical shift predictions using Density Functional Theory (DFT) and Gauge-Including Atomic Orbitals (GIAO). Compounds **1**, **2**, and **4** have two stereocenters, the C-6 spiro-center, which was prominent for all metabolites with the discorhabdins backbone, and a C-3 hydroxyl. As 2D Nuclear Overhauser Effect Spectroscopy (NOESY) correlations for **1**, **2**, and **4** from H-3 to methylene signals H-5 were inconclusive for determining relative configurations of C-3, DFT/GIAO-based chemical shift prediction and comparison tools DP4+ and Sorted Training Set (STS) were employed. A comparison of predicted ^13^C shielding tensors for 3S*,6S* and 3S*,6R* for **1**, **2**, and **4**, at a B3LYP-D_3_BJ/TZVP//wB97X-D3/6-31G* level, were compared to the experimental chemical shifts using STS, resulting in a 97%, 100%, and 100% probability of 3S*,6S* relative configuration for **1**, **2**, and **4**, respectively [12]. To increase our confidence in the assigned relative configuration, shielding tenors were additionally predicted at a B3LYP-D_3_BJ/TZVP//B3YLP/6-311+G** level and compared using DP4+, yielding a 94%, 100%, and 100% probability of the three metabolites possessing a 3S*,6S* configuration when incorporating both ^1^H and ^13^C shielding tensors (Figure 5A) [13].

Compounds **5** and **6** have four chiral centers, C-1, C-2, C-6, and C-8, with eight theoretical relative configurations. Analysis of bond angles C-5/C-6/C-20 and C-1/C-6/C-7 of energy-minimized 3D models indicates that only 6R*,8S* or 6S*,8R* configurations possess plausible geometries around C-6, when C-5 and H-8 are on opposite faces of ring E, due to conformational constraints. Similarly, the examination of bond angles of 3D models, centering on C-2, limit H-2 and C-4 to occupying opposite faces of ring G, consistent with 1X, 2S*,6R*,8S* (*X* = *R* or *S*) configurations (Figure 5A).

**Figure 6:**
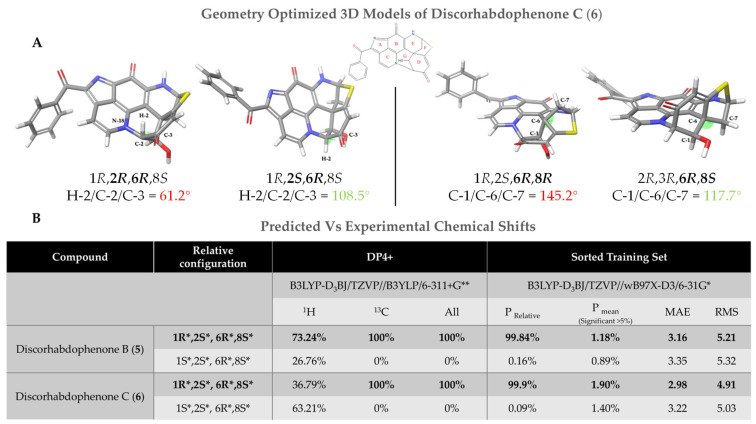
(**A**) Geometry-optimized models of possible relative configurations of **6**, used to determine the bond angles and feasibility of relative configurations. (**B**) DP4+ and STS probabilities from comparing experimental and DFT-predicted chemical shift in relative configurations of compounds **5** and **6**, with results indicating a 1R*,2S*,6R*,8S* configuration.

Examination of coupling constants between H-1/H-2 was inconclusive for the assignment of C-1 relative configuration, with ^3^*J*_HH_ 2.4 Hz being consistent with a 64° dihedral angle, compared to 45° found for 1R*, 2R* and 82° found for 1S*, 2R*, respectively. To determine the relative configuration of C-1, a comparison of DFT/GAIO-based chemical shift predictions and experimental was conducted for 1R*,2S*,6R*,8S* and 1S*,2S*,6R*,8S*, using the same methodology that was applied for **1**, **2**, and **4**. STS and the DP4+ probability calculator predicted >99% and 100% of 1R*,2S*,6R*,8S* relative configurations for both **5** and **6,** respectively (Figure 6B).

#### 2.2.2. Absolute Stereochemistry

**Figure 7:**
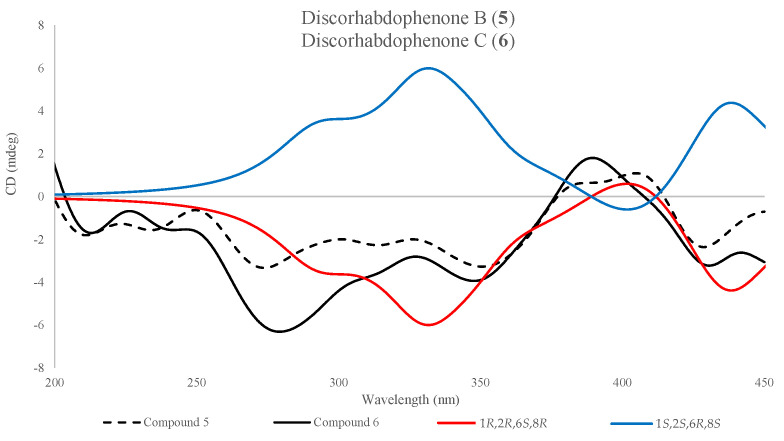
Comparison of observed ECD spectra of discorhabdophenone B (**5**, dashed-black) and discorhabdophenone C (**6**, solid-black) to the TD-DFT-predicted spectra of both epimers of **6**, 1*R*,2*S*,6*R*,8*S* (blue) and 1*S*,2*R*,6*S*,8*R* (red). Spectra were predicted and measured in methanol.

The absolute configuration of compounds **1**, **2**, and **4**–**6** was determined by a comparison of experimental Electronic Circular Dichroism (ECD) and simulated ECD spectra using time-dependent density functional theory (TD-DFT), predicted at a B3LYP-D_3_/CC-PVDZ// B3LYP-D_3_/CC-PVDZ level using a PCM solvation model. Compounds **5** and **6** were assigned an absolute configuration of 1*S*,2*R*,6*S*,8*R* (Figure 7), while **1**, **2**, and **4** were assigned 3*R*,6*R* (Figure 5B). It is worth noting that the predicted spectra of **1** and **2** required a scaling factor of 0.65 to accurately match the experimental ECD spectra.

### 2.3. Bioactivity

The three crude extracts from specimens of *Latrunculia* spp. showed strong cytotoxic properties against a strain of human lung cells (A549) but no activity against *Candida albicans* nor *C. auras*. Upon the purification of metabolites, each were screened for their cytotoxic properties. The new discorhabdin derivatives **1** and **2** showed strong cytotoxicity, with IC_50_’s (Table 3) of 4.3 μM and 1.8 μM, respectively, and the known metabolite (+)-debromodiscorhabdin A (**3**) showed an IC_50_ of 1.0 μM. No biological activity has been previously reported for (+)-debromodiscorhabdin A. Weak cytotoxicity was shown for **4**, with an IC_50_ of 23.9 μM (Table 3), while **5** and **6** were inactive in the cytotoxicity assay. Their activity profile suggested that the discorhabdin backbone had a strong influence on cytotoxicity, with a decrease in cytotoxicity for **4** which had a Δ^16^ olefin compared to the Δ^7^ olefins shown for **1** and **2**. Additionally, **4** had a benzophenone substituent. The benzophenone substituent was also present in **5** and **6** but neither showed cytotoxicity when tested at 50 μg/mL. Compounds **5** and **6** differed from **4** in terms of the C-2/N-18 bridge, a C-5/C-8 sulphur bridge, and Δ^4,16^ olefins. Such structural insights may shed light on a cytotoxicity-optimized derivative.

## 3. Materials and Methods

### 3.1. General Experimental Procedures

Optical rotations were measured using an AutoPol IV digital polarimeter (Hackettstown, NJ, USA) at 589 nm with a 1 dm path length cell. UV-Vis spectra were extracted from HPLC chromatograms. NMR spectra were acquired using a Bruker Neo 600 MHz broadband spectrophotometer (Rheinstetten, Germany). The residual solvent peaks were used as an internal chemical shift reference ((CD_3_)_2_SO: δ_C_ 39.5; δ_H_ 2.50). High-resolution mass spectrometry–liquid chromatography data were obtained on an Agilent 6540 LC-MS QTOF (Wilmington, DE, USA) coupled to an Agilent Jet-stream electrospray ionization detector. H_2_O + 0.1% FA (A) and CH_3_CN + 0.1% FA (B) were used as mobile phases on a Phenomenex Kinetex C_18_ column (2.6 μm, 100 Å, 150 × 3 mm: 0.5 mL/min) (Torrance, CA, USA). Reverse-phase HPLC was performed on a Shimadzu LC20-AT system (Canby, OR, USA) equipped with a photodiode array detector (M20A) using a preparative Phenomenex C_18_ column (5 μm, 100 Å, 250 × 21.2 mm: 10 mL/min) or a semi-preparative Phenomenex C_18_ column (10 μm, 100 Å, 250 × 10 mm: 4 mL/min). CCS values were measured on an Agilent 6560c LC-IMS-Q-TOF (Wilmington, DE, USA) using high-purity N_2,_ with CCS values referenced to Agilent ESI tune Mix, in positive mode, with CCS value calculations using Agilent Mass Hunter IM-MS Browser (build 10.0.1.10039). The drift gas temperature was 27 °C at 3.940 torr. The electronic field was 18.549 V/cm. The methanol and acetonitrile used for column chromatography were obtained from Fisher Co. (Waltham, MA, USA) and were HPLC grade (>99% purity), while the H_2_O was distilled and filtered. Solvents mixtures are reported as *% v*/*v.*

### 3.2. Biological Materials

Two specimens of *Latrunculia* cf. *biformis* (Figure S50) were collected at 210 m depth from the Kerguelen Plateau, Antarctica (−51.137 S, 71.8269 E), by trawling (sample codes ACE16-49, WAMZ44145; ACE16-51, WAMZ44147), with wet weights of 0.15 kg and 1.5 kg, respectively. An additional specimen (sample code PSC18-41, wet weight: 3.3 kg) of *Latrunculia* sp. (Figure S50) was collected by SCUBA at 40 m depth from Cormorant Island near Palmer station, Antarctica (−64.77551 S; −64.071805 W). A small piece of tissue from the two specimens from the Kerguelen Plateau were extracted for DNA using the Qiagen Blood & Tissue DNeasy kit according to the manufacturer’s instructions. A fragment of the mitochondrial Cytochrome Oxidase I (COI) gene was amplified by PCR using primers from [14], purified enzymatically, and bidirectionally Sanger sequenced at the Australian Genome Research Facility (AGRF, Perth, Australia). Reads were assembled and edited using Geneious Prime v2022.1, and analyzed with other relevant data from NCBI in a Maximum Likelihood framework in IQ-tree [15] implementing ModelFinder [16]. The resulting tree shows the phylogenetic placement of the specimens (Figure S51).

### 3.3. Extraction, Isolation, and Purification

Specimens were kept frozen upon collection and freeze-dried on return to our lab. Upon analysis, the three freeze-dried specimens were crushed and extracted separately in MeOH for 24 h. The extracts were filtered and replaced with fresh MeOH twice. Extracts from the three specimens were separately eluted from the HP20 by first washing the fresh resin with Me_2_CO before pre-equilibrating the columns with H_2_O. The extracts were then applied to the resin and eluted from their respective HP20 column before the eluent was diluted with equal volume H_2_O and again eluted from the column. This was repeated a second time. Three fractions of decreasing polarity were created by eluting the column with (A) 30% Me_2_CO/H_2_O, (B) 75% Me_2_CO/H_2_O, and (C) 100% Me_2_CO. Fraction B was further pursued for all three specimens.

Preparative C_18_ HPLC was performed on ACE16-49B (420 mg) with a gradient of 50–100% ACN/H_2_O over 30 min, resulting in 12 fractions, designated B1–B12. The mass spectrum (MS) indicated that fractions B1 (32.4 mg) and B6 (50.2 mg) contained brominated metabolites and therefore they were further prioritized. Fraction B1 was further fractionated by semipreparative C_18_ HPLC with a 10–100% MeOH/H_2_O gradient over 30 min, resulting in the pure compound B1-B. B1-B (**2**: 10 mg, RT = 13.7 min) and B6 (**1**: 50.2 mg, RT = 23.4 min) were determined pure compounds by ^1^H NMR spectroscopy. ACE16-51B (8.3 g) and PSC18-41B (6.2 g) were purified by RP-MPLC with a gradient of 10–100% MeOH/H_2_O over 60 min, resulting in 12 fractions each, designated as B1-B12. Fractions B2, B7, and B8 from ACE16-51 were further fractionated by semipreparative C_18_ HPLC with a gradient of 30–100% ACN/H_2_O (0.1% FA), resulting in the pure compounds (+)-debromodiscorhabdin A (**3**: 2.4 mg, RT = 12.1 min) and discorhabdophenone A (**4**: 6.3 mg, RT = 16.5 min), determined pure by ^1^H NMR spectroscopy. MPLC fractions B6, B7, and B8 from PSC18-41 were all combined due to similarities in ^1^H NMR and MS spectra. They were fractionated by semipreparative C_18_ HPLC with a gradient of 20–100% ACN/H_2_O (0.1% FA) over 30 min, resulting in the pure compounds discorhabdophenone B (**5**: 4.5 mg, RT = 7 min) and discorhabdophenone C (**6**: 1.9 mg, RT = 18.2 min).

### 3.4. Spectroscopic Data (**1**, **2,** and **4**–**6**)

*Discorhabdin* G2 (**1**): deep-red solid; ${\left[\alpha \right]}_{D}^{22}$ −241.4 (*c* 0.029, MeOH); UV (MeOH/H_2_O) λ_max_ (log ε) 211, 228 (sh), 463 (sh) nm (8.22, 8.53, 1.65); ^1^H NMR (600 MHz) and ^13^C NMR (150 MHz) data, see Table 1 and Table S1; HRESIMS *m*/*z* 463.9450 [M + H]^+^ (calcd for C_18_H_14_O_2_N_3_^79^Br, 463.9427; ∆ 4.96 ppm). CCS [M + H]^+^ 195.8 Å2.

*Discorhabdin G3* (**2**): green solid; ${\left[\alpha \right]}_{D}^{22}\;$+207.4 (*c* 0.068, MeOH); UV (MeOH/H_2_O λ_max_ (log ε) 207, 247 (sh), 336 (sh), 564 (sh) nm (7.45, 5.99, 2.79, 0.34); ^1^H NMR (400 MHz) and ^13^C NMR (100 MHz) data, see Table 1 and Table S2; HRESIMS *m*/*z* 386.0505 [M + H]^+^ (calcd for C_18_H_17_O_2_N_3_^79^Br_2_, 386.0499; ∆ 1.6 ppm). CCS [M + H]^+^ 193.0 Å2.

*(+)-Debromodiscorhabdin A* (**3**) was dereplicated by Small Molecular Accurate Recognition Technology (SMART) NMR, with a cosine score of 0.890, by the use of its HSQC data [17]. Compound **3** was identified by its multiplicity-edited HSQC data as having three olefinic protons, two deshielded methines, and four methylene groups. Its HRESIMS displayed an [M + H]^+^ ion at *m*/*z* 338.0976, which, with its ^1^H and ^13^C NMR data (see Table S3), matches the molecular formula of C_18_H_16_N_3_O_2_S for the known debromodiscorhabdin A (**3**) [7]. The published optical rotation of ${\left[\alpha \right]}_{D}^{22}\;$+207 agrees well the experimental optical rotation of ${\left[\alpha \right]}_{D}^{22}\;$+178.9.

*Discorhabdophenone A* (**4**): deep-red solid; ${\left[\alpha \right]}_{D}^{22}\;$+66.0 (*c* 0.076, MeOH); UV (ACN/H_2_O) λ_max_ (log ε) 229, 306 (sh), 462 (sh) nm (21.96, 11.67, 4.03); ^1^H NMR (400 MHz) and ^13^C NMR (100 MHz) data, see Table 2 and Table S4; HRESIMS *m*/*z* 490.0773 [M + H]^+^ (calcd for C_25_H_21_O_3_N_3_^79^Br, 490.0761; Δ 2.5 ppm). CCS [M + H]^+^ 214.1 Å2.

*Discorhabdophenone B* (**5**): deep-red solid; ${\left[\alpha \right]}_{D}^{22}\;$+310.5 (*c* 0.11, MeOH); UV (ACN/H_2_O) λ_max_ (log ε) 214, 282 (sh), 469 (sh) nm (19.16, 14.65, 3.68); ^1^H NMR (400 MHz) and ^13^C NMR (100 MHz) data, see Table 2 and Table S5; HRESIMS *m*/*z* 470.0810 [M + H]^+^ (calcd for C_25_H_16_O_5_N_3_S, 470.0805; Δ 1.1 ppm). CCS [M + H]^+^ 208.2 Å2.

*Discorhabdophenone C* (**6**): deep-red solid; ${\left[\alpha \right]}_{D}^{22}\;$+229.2 (*c* 0.096, MeOH); UV (ACN/H_2_O) λ_max_ (log ε) 205, 282 (sh), 475 (sh) nm (10.11, 6.76, 1.97); ^1^H NMR (400 MHz) and ^13^C NMR (100 MHz) data, see Table 2 and Table S6; HRESIMS *m*/*z* 454.0861 [M + H]^+^ (calcd for C_25_H_16_O_4_N_3_S, 454.0856; Δ 1.1 ppm). CCS [M + H]^+^ 208.1 Å2.

### 3.5. Computational Methods

All molecular mechanics and quantum mechanics calculations were integrated using Macromodel and Jaguar (version 2024-1, Schrodinger LLC) and ORCA 6.0.1 [18,19,20,21,22,23].

#### 3.5.1. Conformer Generation

Conformation searches for each isomer utilized OPLS4 to generate low-energy conformers within a 5 kcal/mol energy window, in the liquid phase (water), using a mixed torsional/low-mode sampling approach. Conformers with <0.5 Å atom deviation were removed to prevent redundancy.

#### 3.5.2. Chemical Shift and Shielding Tensor Predictions

Conformers underwent geometry optimization using DFT at the B3LYP-D3(BJ)/TZVP level with a CPCM (DMSO) solvation model and tightSCF convergence tolerance using ORCA 6.0.1. Frequency calculations were conducted on geometry-optimized conformers and conformers with a negative frequency were re-optimized with an extremeSCF convergence tolerance. NMR shielding tensors were predicted using DFT/GIAO at the ωB97X-D/6-31G* level, with bromine atoms being calculated at the ωB97X-D/6-311G* level, using a CPCM solvation model (DMSO), for use with the Sorted Training Set (STS) comparison tool. Shielding tensor predictions were repeated at a B3YLP/6-311+G** level for use with the DP4+ probability predictor. Boltzmann-weighted shielding tensors were calculated based on each conformers Gibbs Free energy calculated at B3LYP-D3(BJ)/TZVP level. A Sorted Training Set (STS) excel sheet was used to compare the experimental ^13^C chemical shift to predicted chemical shifts for each configuration. Additionally, a DP4+ excel sheet was used for a comparison of experimental ^1^H and ^13^C chemical shifts to predicted shielding tensors. The experimental and predicted chemical shifts in the non-equivalent protons were aligned based on their relative shielding, with upfield and downfield trends used to guide the assignments. Exchangeable protons were excluded from chemical shift comparisons. Metabolites were calculated as neutral molecules.

#### 3.5.3. Electronic Circular Dichroism Spectral Predictions

Conformers underwent geometry optimization and subsequent relative thermal free energies (ΔG) at 298.15 K, using DFT at the B3LYP-D3/cc-pVDZ level. Geometry optimization was carried out using a methanol PCM solvation model, while single-point energy calculations were conducted using a PBF solvent model for improved energy calculation accuracy. Conformers with negative vibrational frequencies were removed. ECD spectra for each conformer were computed using TD-DFT at the B3LYP-D3/cc-pVDZ level, using 20 excited states generated by Tamm–Dancoff approximation. Boltzmann conformer populations were used to create a weighted averaged ECD spectrum. Experimental and predicted spectra were visualized using Excel and a Schrodinger spectral plot tool. A comparison between experimental and predicted UV spectra was used to determine wavelength corrections. Predicted ECD spectra were normalized to match the intensities of experimental signals. Metabolites were calculated as neutral molecules. The predicted spectra of **1** and **2** required a scaling factor of 0.65 to accurately match the experimental ECD spectra.

### 3.6. Cytotoxicity Assay

Stock solutions of A549 cells were prepared at 1 × 10^4^ cells/mL in growth media (FK12 medium Kaighn’s modification, 10% bovine growth serum, 1% penicillin-streptomycin) using a calibration curve based on UV-Vis absorbance at 450, 530, and 600 nm. To each well of a 96-well plate 200 μL of this solution was added and incubated for 24 h at 37 °C and 5% CO_2_ atmosphere. Each testing solution (2 μL) was then added to the wells in triplicate and the plate was incubated for 72 h, at which point the media in each plate was removed and each well was washed with 1× phosphate-buffered saline (PBS). After the solution of MTT (1.25 mg/mL in 75% growth media 25% PBS) was added to each well, the plate was incubated. After 4 h of incubation, the media was removed carefully to avoid disturbing the crystals in the bottom of each well. DMSO (200 μL) was added to each well and the cell viability was calculated as a percentage using the negative control. DMSO was used as a negative control, and the positive control was nystatin.

## 4. Conclusions

Three specimens of *Latrunculia* spp. from two different Southern Ocean locations were found with new discorhabdin derivatives, including the novel C-14 phenone function. The highly colored metabolites were purified using RP-HPLC by targeting characteristic ^1^H NMR signals and masses that sometimes displayed isotopic patterns. Five unreported metabolites were isolated, **1**, **2,** and **5**–**6**, as well as one previously described metabolite, **3**. Their cytotoxic properties were investigated against the A549 cell lines, where **1** and **2** displayed significant cytotoxicity at 4.3 μM and 1.8 μM, respectively. Compounds **4**–**6** showed weak or no cytotoxicity and differ from the other discorhabdins by the aforementioned phenone substituent.

## Figures and Tables

**Table 1:** NMR data for Discorhabdin G2 and Discorhabdin G3 (**1** and **2**) (600 (^1^H) and 150 (^13^C) MHz, (CD_3_)_2_SO)).

Pos.	1				2	
	δ_C_, Type	δ_H_ (*J* in Hz)	gCOSY	gHMBC	δ_C_, Type	δ_H_ (*J* in Hz)
**1**	134.9, CH	6.15, s		2, 3, 5, 6, 7, 20	134.7, CH	6.33, s
**2**	129.2, C				132.1, C	
**3**	67.3, CH	4.42, t (7.1)	4a, 4b	1, 2, 4	67.2, CH	4.42, t (7.8)
**4a**	32.7, CH_2_	2.18, qt (13.1, 4.4)	3, 4b, 5a	2, 3, 5, 6	27.4, CH_2_	1.91, dt (7.3, 3.0)
**4b**		2.04, dq (4.9, 7.5)	3, 4a	2, 3, 5, 6		
**5a**	36.9, CH_2_	2.70, td (13.6, 4.0)	4a, 5b	3, 4, 6, 7, 20	34.9, CH_2_	2.27, m
**5b**		2.11, dt (14.0, 3.6)		1, 3, 4, 6, 7, 20		1.84, d (13.1)
**6**	45.9, C				40.3, C	
**7**	104.0, C				115.4, CH	5.24, d (7.6)
**8**	125.5, CH	6.60, d (5.3)	9	6, 7, 10	121.8, CH	6.28, dd (3.1, 4.5)
**9**		8.72, d (4.2)	8	7, 11, 20		10.43, s
**10**	137.9, C				144.6, C	
**11**	164.1, C				166.4, C	
**12**	118.5, C				123.4, C	
**13**						13.17, s
**14**	128.6, CH	8.24, s		11, 12, 15, 21	126.8, CH	7.36, s
**15**	124.6, C				119.4, C	
**16**	113.7, CH	7.54, d (5.8)	17	14, 17, 19, 21	17.9, CH_2_	2.87, t (7.5)
**17**	141.6, CH	8.31, d (5.8)	16	15, 16, 19, 21	44.8, CH_2_	3.91, dd (5.9, 7.1)
**18**						8.78, s
**19**	147.3, C				157.2, C	
**20**	111.7, C				99.6, C	
**21**	119.5, C				122.4, C	

**Table 2:** ^1^H and ^13^C NMR data for Discorhabdophenone A, B, and C (**4–6**) ((600 (^1^H) and 150 (^13^C) MHz, (CD_3_)_2_SO)).

Pos.	4		5		6	
	δ_C_, Type	δ_H_ (*J* in Hz)	δ_C_, Type	δ_H_ (*J* in Hz)	δ_C_, Type	δ_H_ (*J* in Hz)
**1**	137.3, CH		67.2, CH	4.84, s	67.2, CH	4.85, t (1.8)
**2**	132.8, C		65.7, CH	5.02, s	65.7, CH	5.04, d (2.4)
**3**	68.0, CH	4.59, br s	184.5, C		184.5, C	
**4a**	29.6, CH_2_	2.06, o/l	109.9, CH	5.96, s	110.0, CH	5.97, s
**4b**		1.90, o/l				
**5**	31.1, CH_2_	2.10, o/l	171.3, CH		171.2, C	
		1.90, o/l				
**6**	38.7, C		47.5, C		47.5, C	
**7a**	31.1, CH_2_	2.06, o/l	36.0, CH_2_	2.86, d (10.9)	36.0, CH_2_	2.86, dd (3.6, 10.7)
**7b**		1.63, td (3.1, 12.6)		2.61, d (11.2)		2.61, d (11.8)
**8a**	37.5, CH_2_	3.55, dt (3.1, 13.3)	63.4, CH	5.70, s	63.4, CH	5.71, d (2.0)
**8b**		3.32, o/l				
**9**				9.30, s		9.34, s
**10**	145.6, C		145.5, C		145.2, C	
**11**	169.2, C		168.7, C		169.1, C	
**12**	133.2, C		132.3, C		133.2, C	
**14**	151.0, C		130.8, C		130.7, C	
**15**	122.1, C		121.1, C		121.3, C	
**16**	112.0, CH	7.84, d (5.7)	115.1, CH_2_	7.94, (6.5)	114.9, CH	7.97, d (6.7)
**17**	129.9, CH	7.91, d (5.7)	131.5, CH_2_	8.05, (6.4)	131.9, CH	8.10, d (6.5)
**18**						
**19**	139.2, C		146.0, C		145.4, C	
**20**	101.2, C		102.2, C		102.4, C	
**21**	132.1, C		132.7, C		132.5, C	
**22**	189.1, C		186.2, C		188.2, C	
**23**	138.9, C		129.2, C		138.0, C	
**24**	130.8, CH	8.37, d (5.6)	133.1, CH	8.42, d (8.2)	130.4, CH	8.35, d (7.8)
**25**	128.4, CH	7.56, t (7.5)	114.8, CH	6.90, d (8.2)	128.0, CH	7.55, t (7.6)
**26**	132.3, CH	7.63, t (6.9)	161.5, C		132.1, CH	7.63, t (7.4)

**Table 3:** Cytotoxicity activity against the A549 cells (IC_50_).

Compound	IC_50_ (μM)	SD ^a^ (µM)
**1**	4.3	1.1
**2**	1.8	0.1
**3**	1.0	0.4
**4**	23.9	0.3
**5**	>50	ND ^b^
**6**	>50	ND ^b^

a = standard deviation; b = not determined.

## Data Availability

The NMR data for the following compounds have been deposited in the Natural Products Magnetic Resonance Database (NP-MRD; www.np-mrd.org, accessed on 30 July 2025 and can be found at NP0351379 (Discorhabdin G2), NP0351380 (Discorhabdin G3), NP0351381 (Discorhabdophenone A), NP0351382 (Discorhabdophenone B), and NP NP0351383 (Discorhabdophenone C). Other data not found in the Supplementary Materials will be available upon request to the corresponding author.

## Supplementary Materials

The following supporting information can be downloaded at https://www.mdpi.com/article/10.3390/md23100401/s1. Figures S1–S51 and Tables S1–S6: ^1^H, ^13^C, COSY, HSQC, and HMBC NMR spectra, as well as HRMS, and geometry-optimized conformers of metabolites **1**, **2,** and **4**–**6**; maximum likelihood tree of Latrunculiidae COI; photographs of study specimens.
