# Supplementary material for: Novel Discorhabdin Derivatives from Antarctic Sponges of the Genus Latrunculia: Expanding the Chemical Diversity of Polar Marine Natural Products
Source: Mar Drugs. 2025 Oct 15;23(10):401. doi: 10.3390/md23100401 (PMC12565582; doi:10.3390/md23100401)
Supplement: Supplementary file 1 [file marinedrugs-23-00401-s001.zip › marinedrugs-3834282 SI.pdf]

Supporting Information

Novel Discorhabdin Derivatives from Antarctic Sponges of the Genus *Latrunculia*: Expanding the Chemical Diversity of Polar Marine Natural Products

Sam Afoullouss, Stine S. H. Olsen, Sydney Morrow, Ezequiel Cruz Rosa, Kaley Geu, Nerida G. Wilson and Bill J. Baker

Table of Content

Table S1. NMR data for **1** (600 (^{1}H) and 150 (^{13}C) MHz, $(CD_3)_2SO$) 3
Figure S1. Structure of **1**. 3
Figure S2. Full scale (0.0-16.0 ppm) ^{1}H NMR spectrum (600 MHz, $(CD_3)_2SO$) of **1** inset with 2.0-2.8 ppm. 4
Figure S3. Full scale BB ^{13}C NMR spectrum (150 MHz, $(CD_3)_2SO$) of **1**. 4
Figure S4. COSY NMR spectrum (600 MHz, $(CD_3)_2SO$) of **1**. 5
Figure S5. HSQC-DEPT NMR spectrum (600 MHz, $(CD_3)_2SO$) of **1**. 5
Figure S6. HMBC NMR spectrum (600 MHz, $(CD_3)_2SO$) of **1**. 6
Figure S7. NOESY NMR spectrum (600 MHz, $(CD_3)_2SO$) of **1**. 6
Figure S8. HRESIMS analysis of **1**. 6
Table S2. NMR data for (**2**) (600 (^{1}H) and 150 (^{13}C) MHz, $(CD_3)_2SO$). 7
Figure S9. Structure of **2**. 7
Figure S10. Full scale (0.0-14.0 ppm) ^{1}H NMR spectrum (600 MHz, $(CD_3)_2SO$) of **2**. 8
Figure S11. Full scale BB ^{13}C NMR spectrum (150 MHz, $(CD_3)_2SO$) of **2** inset with 121.5-123.5 ppm. 8
Figure S12. COSY NMR spectrum (600 MHz, $(CD_3)_2SO$) of **2**. 9
Figure S13. HSQC-DEPT NMR spectrum (600 MHz, $(CD_3)_2SO$) of **2**. 9
Figure S14. HMBC NMR spectrum (600 MHz, $(CD_3)_2SO$) of **2**. 10
Figure S15. NOESY NMR spectrum (600 MHz, $(CD_3)_2SO$) of **2**. 10
Figure S16. HRESIMS analysis of **2**. 10
Table S3. Comparison of experimental and published NMR data for **3** (600 (^{1}H) and 150 (^{13}C) MHz) in MeOH-d_4 11
Table S4. NMR data for Discorhabdophenone A (**4**) (600 (^{1}H) and 150 (^{13}C) MHz, $(CD_3)_2SO$). 12
Figure S17. Structure of **4**. 12
Figure S18. Full scale (0.0-12 ppm) ^{1}H NMR spectrum (600 MHz, $(CD_3)_2SO$) of **4**. 13
Figure S19. Full spectrum (0-215 ppm) BB ^{13}C NMR spectrum (150 MHz, $(CD_3)_2SO$) of **4**. 13
Figure S20. Expanded (28-190 ppm) BB ^{13}C NMR spectrum (150 MHz, $(CD_3)_2SO$) of **4**. 14
Figure S21. COSY NMR spectrum (600 MHz, $(CD_3)_2SO$) of **4**. 14
Figure S22. HSQC-DEPT NMR spectrum (600 MHz, $(CD_3)_2SO$) of **4**. 15
Figure S23. HMBC NMR spectrum (600 MHz, $(CD_3)_2SO$) of **4**. 15
Figure S24. NOESY NMR spectrum (600 MHz, $(CD_3)_2SO$) of **4**. 16
Figure S25. HRESIMS analysis of **4**. 16
Table S5. NMR data for discorhabdophenone B (**5**) (600 (^{1}H) and 150 (^{13}C) MHz, $(CD_3)_2SO$). 17
Figure S26. Chemical structure of **5**. 17
Figure S27. Full scale (0.0-12.0 ppm) ^{1}H NMR spectrum (600 MHz, $(CD_3)_2SO$) of **5**. 18
Figure S28. Expanded (2.0-10.5 ppm) ^{1}H NMR spectrum (600 MHz, $(CD_3)_2SO$) of **5**. 18
Figure S29. Full scale (-20.0-220.0 ppm) DEPTQ135 NMR spectrum (150 MHz, $(CD_3)_2SO$) of **5**. 19
Figure S30. Expanded (30.0-190.0 ppm) DEPTQ135 NMR spectrum (150 MHz, $(CD_3)_2SO$) of **5**. 19
Figure S31. COSY NMR spectrum (600 MHz, $(CD_3)_2SO$) of **5**. 20
Figure S32. TOCSY NMR spectrum (600 MHz, (CD3)2SO) of **5**. 20
Figure S33. HSQC-DEPT NMR spectrum (600 MHz, $(CD_3)_2SO$) of **5** with the DEPTQ spectrum on F1 axis. 21
Figure S34. HMBC NMR spectrum (600 MHz, $(CD_3)_2SO$) of **5** with DEPTQ spectrum on F1 axis. 21
Figure S35. NOESY NMR spectrum (600 MHz, $(CD_3)_2SO$) of **5**. 22
Figure S36. HRESIMS analysis of **5**. 22
Figure S37. Analysis of the protonation state of **5**. 22
Table S6. NMR data for discorhabdophenone C (**6**) (600 (^{1}H) and 150 (^{13}C) MHz, $(CD_3)_2SO$). 23
Figure S38. Chemical structure of **6**. 23
Figure S39. Full scale (0.0-12.0 ppm) ^{1}H NMR spectrum (600 MHz, $(CD_3)_2SO$) of **6**. 24
Figure S40. Expanded (2.0-9.5 ppm) ^{1}H NMR spectrum (600 MHz, $(CD_3)_2SO$) of **6** inset with 7.54-7.64 ppm. 24
Figure S41. Full scale (-20.0-220.0 ppm) DEPTQ135 NMR spectrum (150 MHz, $(CD_3)_2SO$) of **6**. 25
Figure S42. Expanded (30.0-190.0 ppm) DEPTQ135 NMR spectrum (150 MHz, $(CD_3)_2SO$) of **6**. 25
Figure S43. COSY NMR spectrum (600 MHz, $(CD_3)_2SO$) of **6**. 26
Figure S44. TOCSY NMR spectrum (600 MHz, (CD3)2SO) of **6**. 26
Figure S45. HSQC-DEPT NMR spectrum (600 MHz, $(CD_3)_2SO$) of **6** with DEPTQ135 on F1 axis. 27
Figure S46. HMBC NMR spectrum (600 MHz, $(CD_3)_2SO$) of **6**. 27

Figure S47. NOESY NMR spectrum (600 MHz, $(CD_3)_2SO$) of **6**. 28
Figure S48. HRESIMS analysis of **6**. 28
Figure S49. Analysis of the protonation state of **6**. 28
Figure S50. Photo documentation of specimens. Top: Voucher photos of ACE16 specimens. Bottom: In situ (Cormorant Island, 125', 13 Mar 2018) and voucher photos (scale bar is 15 cm) of PSC18 specimen. 29
Figure S51. Maximum likelihood tree of Latrunculiidae COI from new specimens and available data on NCBI. Nodes were tested with 1000 ultrafast bootstraps, values below 50 have been removed, as well as short internode values for readability. 30

Table S1. NMR data for **1** (600 (^{1}H) and 150 (^{13}C) MHz, $(CD_3)_2SO$).

pos	δ_C, type	δ_H (*J* in Hz)	gCOSY	gHMBC	Key NOESY
**1**	134.9, CH	6.15, s		2, 3, 5, 6, 7, 20	3, 5a, 5b
**2**	129.2, C				
**3**	67.3, CH	4.42, dd (8.7, 4.2)	4a, 4b	1, 2, 4	4a, 5a
**4a**	32.7, CH_2	2.18, dddd (13.1, 13.1, 8.7, 4.4)	3, 4b, 5a	2, 3, 5, 6	3
**4b**		2.04, dddd (13.1, 3.9, 3.9, 3.9)	3, 4a	2, 3, 5, 6	
**5a**	36.9, CH_2	2.70, ddd (13.4, 13.4, 4.0)	4a, 5b	3, 4, 6, 7, 20	1, 3
**5b**		2.11, ddd (13.8, 3.9, 3.9)		1, 3, 4, 6, 7, 20	1, 8
**6**	45.9, C				
**7**	104.0, C				
**8**	125.5, CH	6.60, d (5.3)	9	6, 7, 10	5b, 9
**9**		8.72, d (4.2)	8	7, 11, 20	8
**10**	137.9, C				
**11**	164.1, C				
**12**	118.5, C				
**14**	128.6, CH	8.24, s		11, 12, 15, 21	
**15**	124.6, C				
**16**	113.7, CH	7.54, d (5.8)	17	14, 17, 19, 21	17
**17**	141.6, CH	8.31, d (5.8)	16	15, 16, 19, 21	16
**19**	147.3, C				
**20**	111.7, C				
**21**	119.5, C				

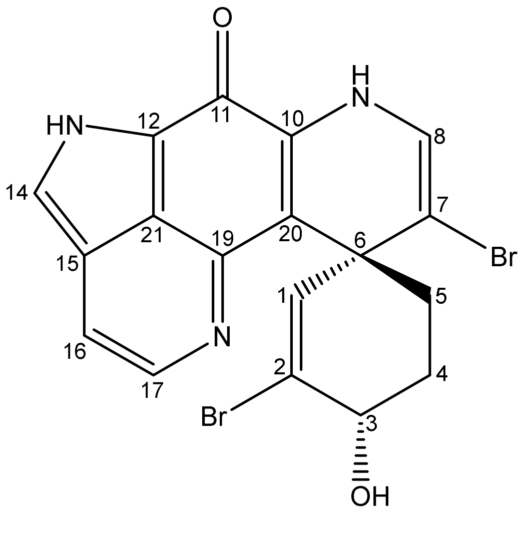

Figure S1. Structure of **1**.

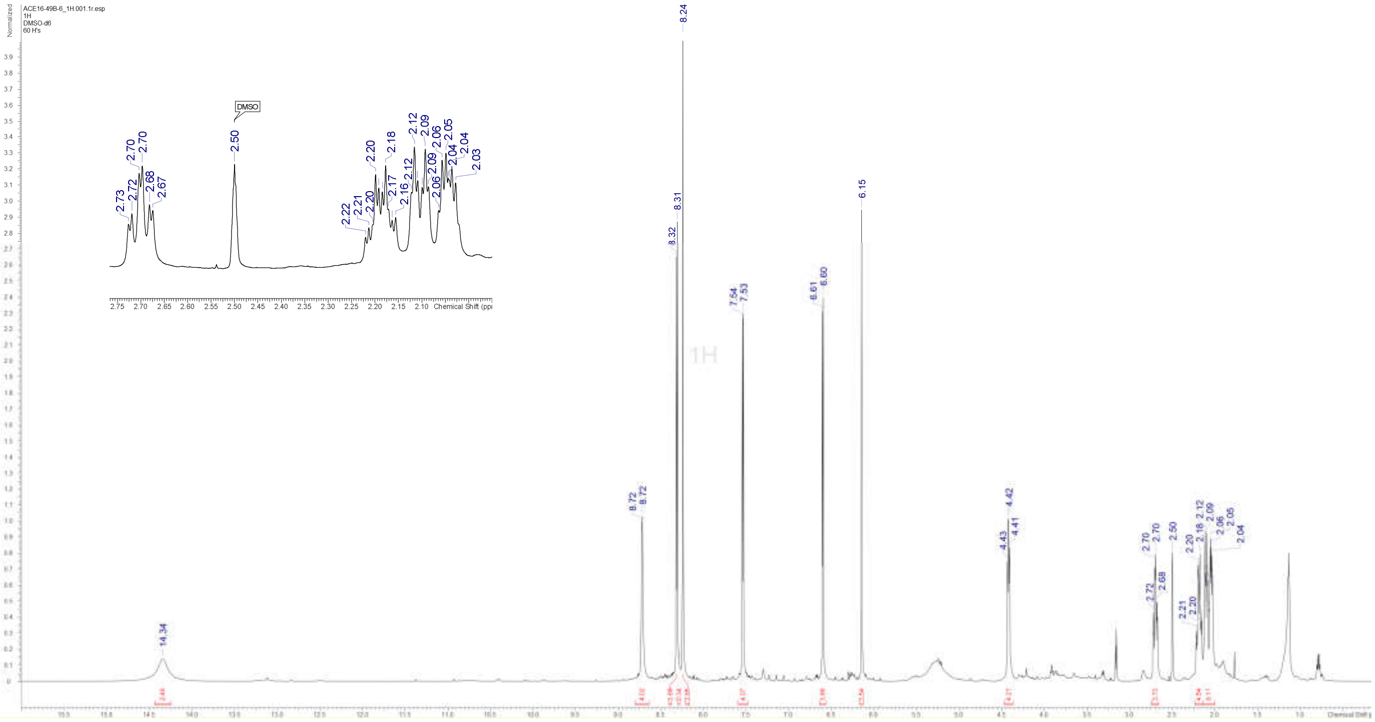

Figure S2. Full scale (0.0-16.0 ppm) ^{1}H NMR spectrum (600 MHz, $(CD_3)_2SO$) of **1** inset with 2.0-2.8 ppm.

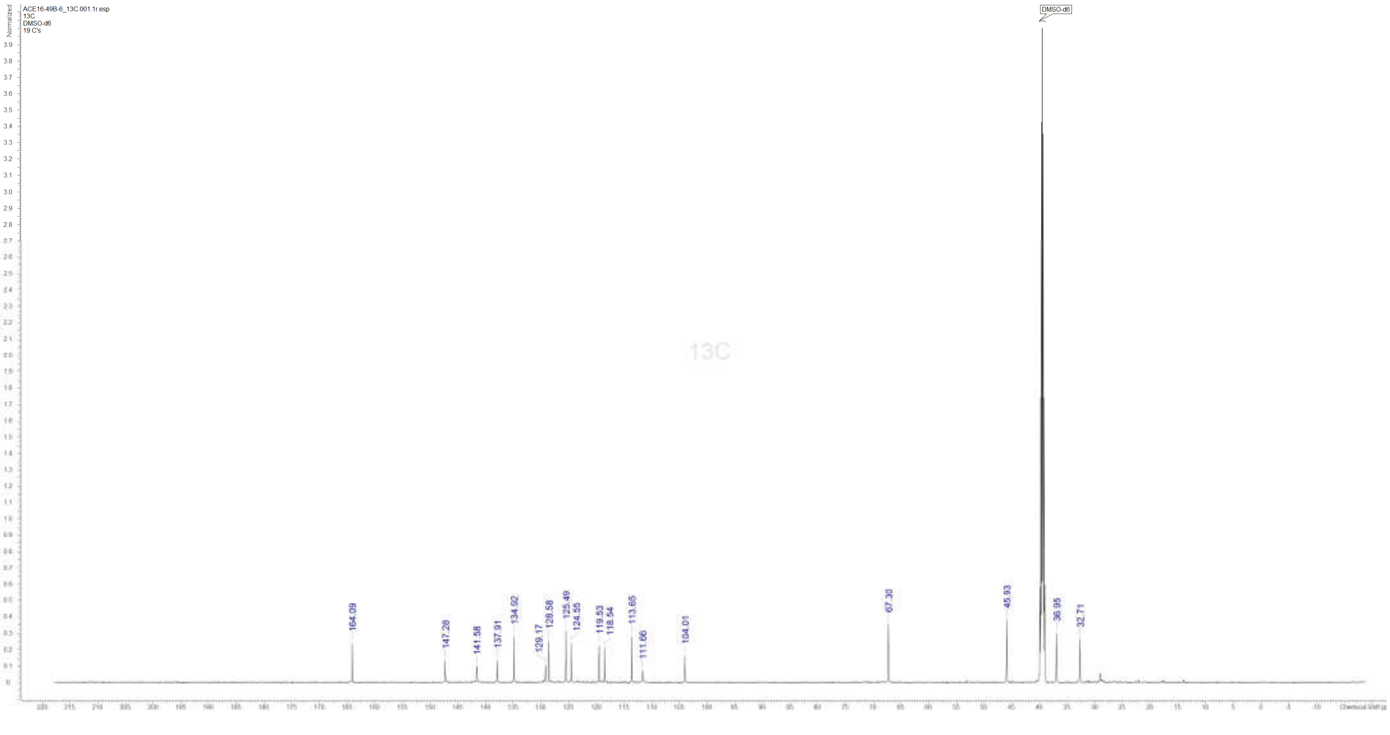

Figure S3. Full scale BB ^{13}C NMR spectrum (150 MHz, $(CD_3)_2SO$) of **1**.

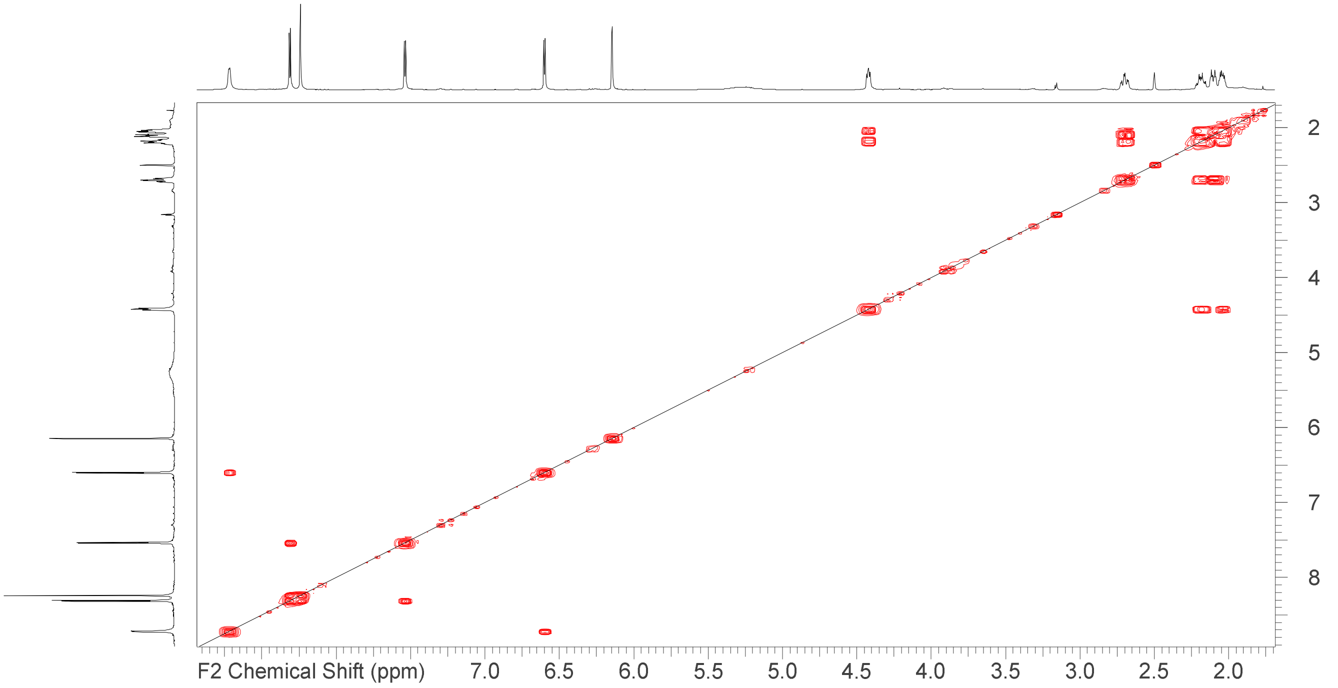

Figure S4. COSY NMR spectrum (600 MHz, $(CD_3)_2SO$) of **1**.

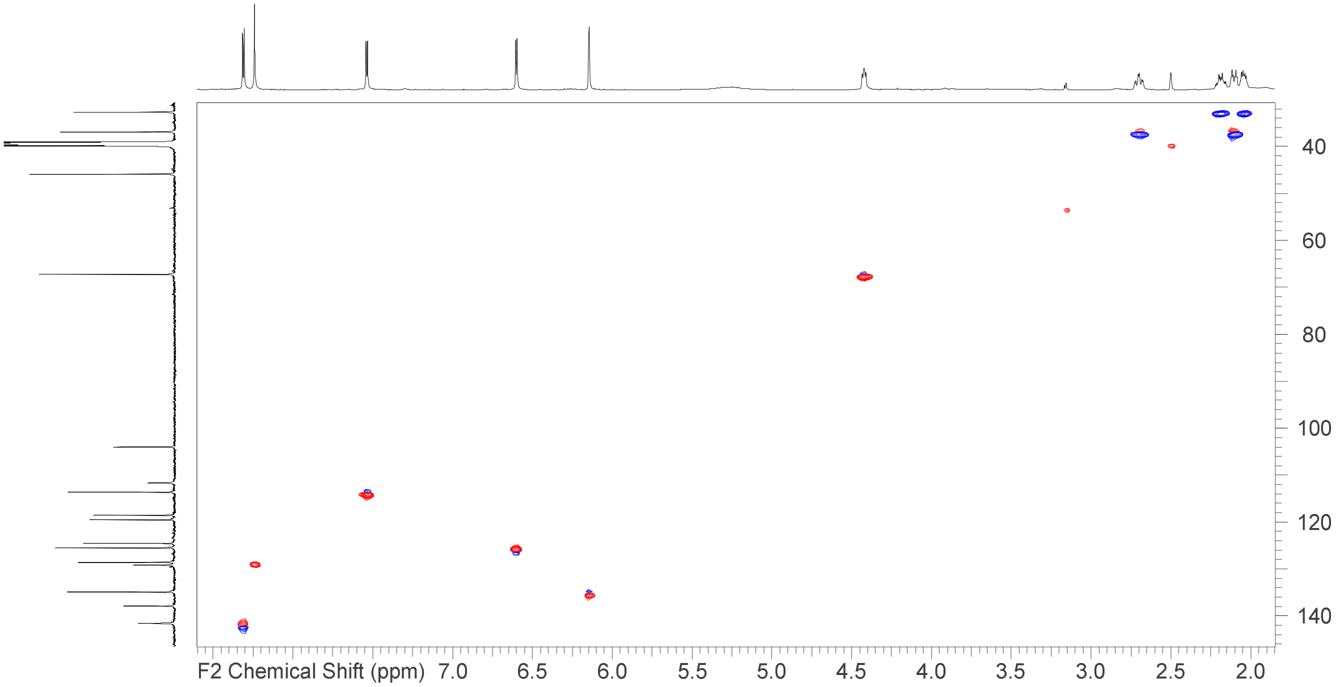

Figure S5. HSQC-DEPT NMR spectrum (600 MHz, $(CD_3)_2SO$) of **1**.

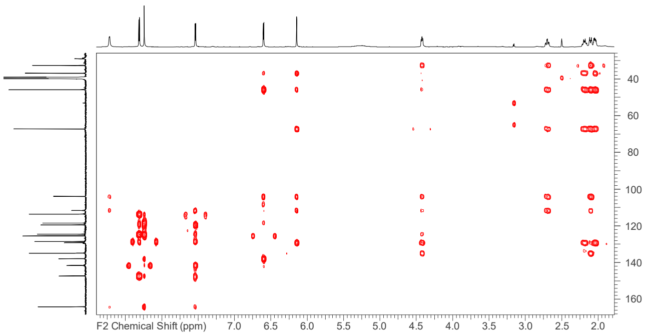

Figure S6. HMBC NMR spectrum (600 MHz, $(CD_3)_2SO$) of **1**.

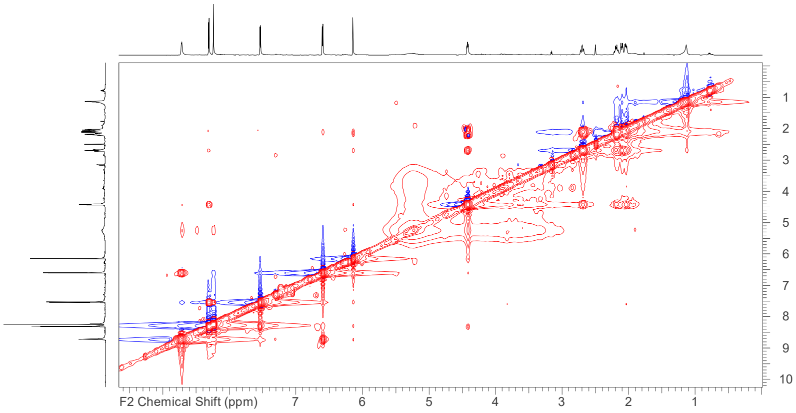

Figure S7. NOESY NMR spectrum (600 MHz, $(CD_3)_2SO$) of **1**.

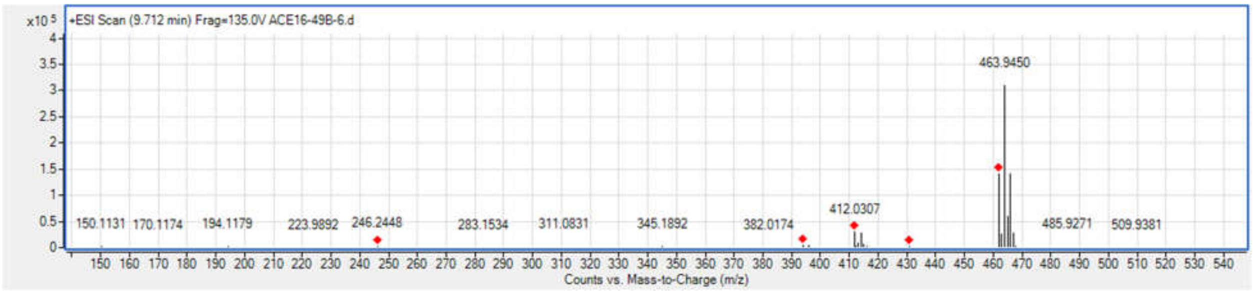

Figure S8. HRESIMS analysis of **1**; $[M + H]^+$: 463.9450 for $C_{18}H_{14}N_3O_2{}^{79}Br^{81}Br$ (calcd 463.9427).

Table S2. NMR data for (**2**) (600 (^{1}H) and 150 (^{13}C) MHz, $(CD_3)_2SO$).

pos	δ_C, type	δ_H (*J* in Hz)	gCOSY	gHMBC	Key NOESY	Key ROESY
**1**	134.7, CH	6.33, s		2, 3, 5, 6, 20	7	7
**2**	132.1, C					
**3**	67.2, CH	4.42, t (7.7)	4	1, 2, 4	4, 5a	4, 5a
**4**	27.4, CH_2	1.91, dt (7.3, 3.0)	3, 5a, 5b	2, 3, 5, 6	3, 5a, 7	
**5a**	34.9, CH_2	2.27, m	4, 5b	4, 6, 7	3, 4, 5b, 18	4, 5b
**5b**		1.84, dt (13.1, 3.2)	4, 5a	1, 3	5a	
**6**	40.3, C					
**7**	115.4, CH	5.24, d (7.6)	8	1, 6, 8, 20	1, 4	4
**8**	121.8, CH	6.28, dd (7.6, 4.5)	7, 9	6, 7, 10		7
**9**		10.43, s	8	20		
**10**	144.6, C					
**11**	166.4, C					
**12**	123.4, C					
**13**		13.17, s	14	15		
**14**	126.8, CH	7.36, d (1.2)	13	11, 12, 15		
**15**	119.4, C					
**16**	17.9, CH_2	2.87, t (7.4)	17	14, 15, 17, 21		
**17**	44.8, CH_2	3.91, dt (7.4, 6.9)	16, 18	15, 16, 19		
**18**		8.78, s	17		5a, 17	5a
**19**	157.2, C					
**20**	99.6, C					
**21**	122.4, C					

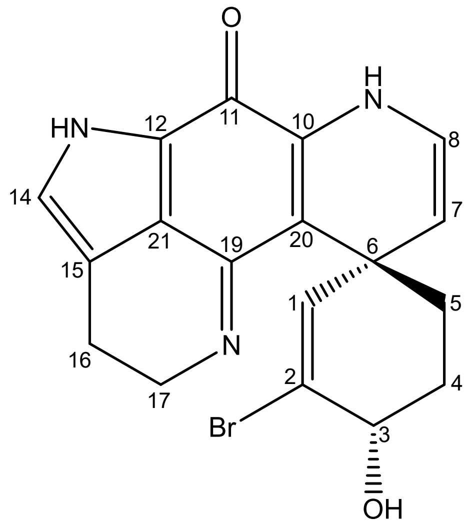

Figure S9. Structure of **2**.

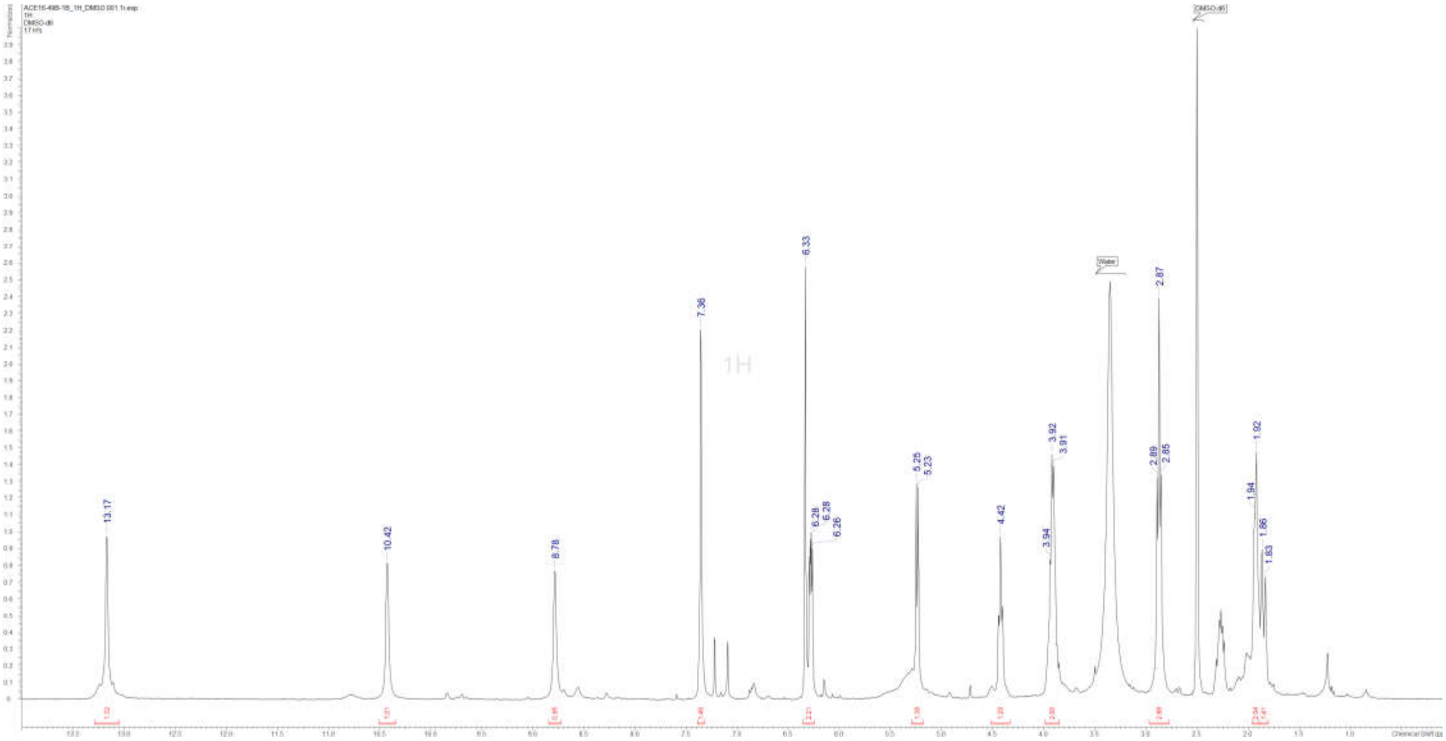

Figure S10. Full scale (0.0-14.0 ppm) ^{1}H NMR spectrum (600 MHz, $(CD_3)_2SO$) of **2**.

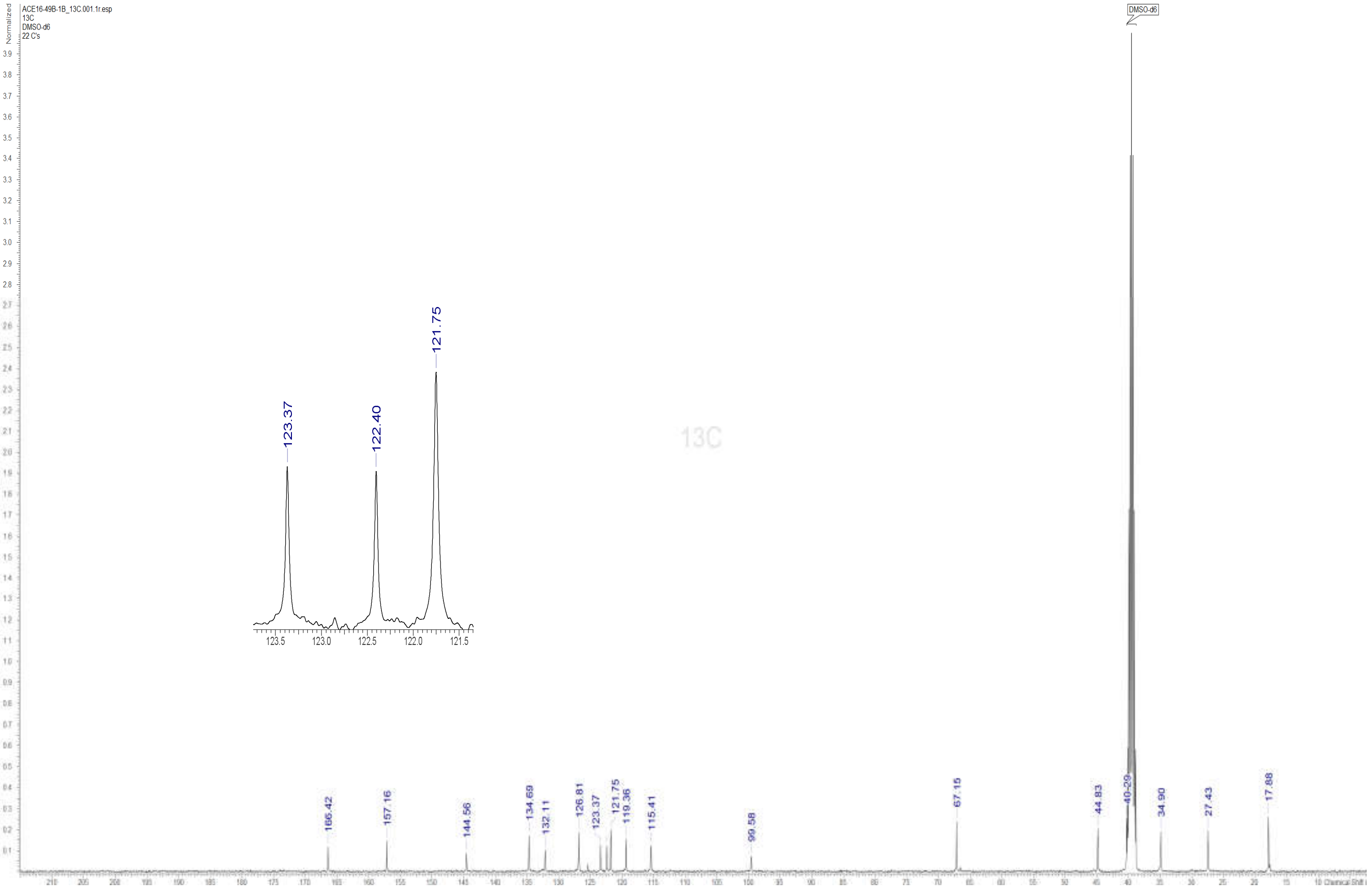

Figure S11. Full scale BB ^{13}C NMR spectrum (150 MHz, $(CD_3)_2SO$) of **2** inset with 121.5-123.5 ppm.

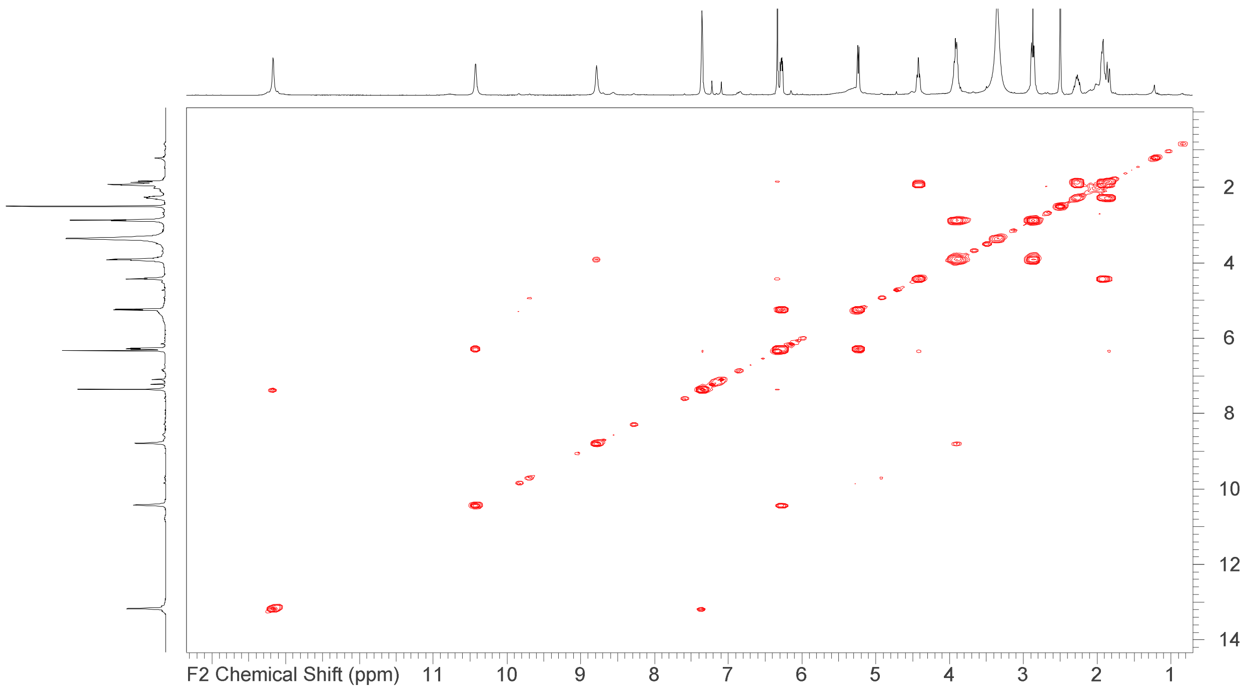

Figure S12. COSY NMR spectrum (600 MHz, $(CD_3)_2SO$) of **2**.

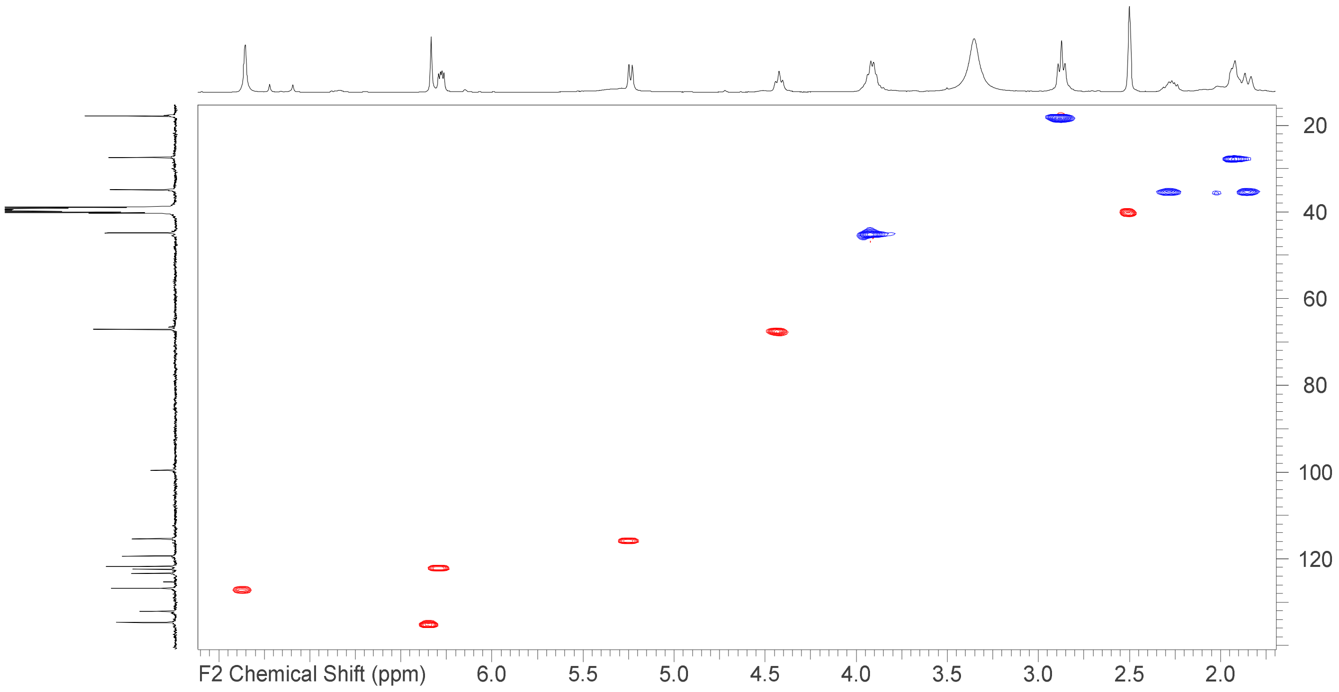

Figure S13. HSQC-DEPT NMR spectrum (600 MHz, $(CD_3)_2SO$) of **2**.

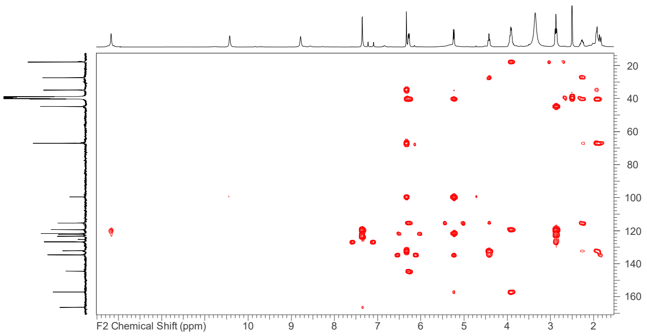

Figure S14. HMBC NMR spectrum (600 MHz, $(CD_3)_2SO$) of **2**.

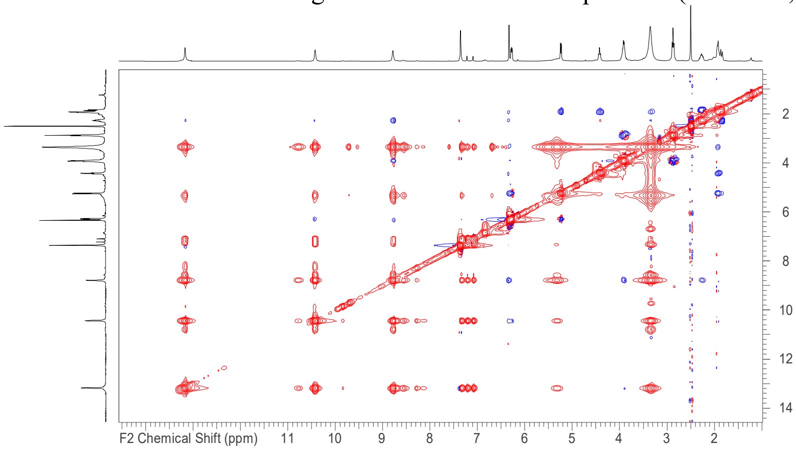

Figure S15. NOESY NMR spectrum (600 MHz, $(CD_3)_2SO$) of **2**.

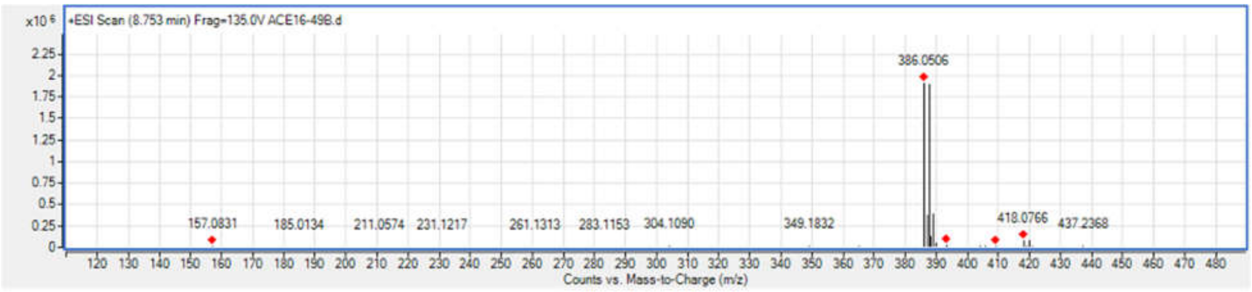

Figure S16. HRESIMS analysis of **2**.

Table S3. Comparison of experimental and published NMR data for **3** (600 (^{1}H) and 150 (^{13}C) MHz) in MeOH-d_4.

	Experimental		Published	
pos	δ_C, type	δ_H (J in Hz)	δ_C, type	δ_H (J in Hz)
**1**	150.1, CH	7.11 (d, 10.2)	150.3, CH	7.11 (d, 10.2)
**2**	132.0, CH	6.31 (d, 10.2)	132.1, CH	6.30 (d, 10.2)
**3**	196.6, C		196.8, C	
**4a**	46.0 CH_2	2.90 (o/l)	46.1, CH_2	2.90 (m)
**4b**		2.67(dd, 6.5, 16.9)		2.67 (dd, 6.7, 17.2)
**5**	54.1, CH_2	4.50 (dd, 6.7, 12.2)	55.8, CH_2	4.49 (dd, 6.7, 12.5)
**6**	n/o		48.3, C	
**7a**	42.4, CH_2	2.91 (o/l)	42.5, CH_2	2.90 (m)
**7b**		2.58 (d, 12.3)		2.58 (d, 12.4)
**8**	60.3, CH	5.36 (dd, 3.9, 3.9)	60.4, CH	5.36 (d, 3.5)
**10**	153.0		153.0, C	
**11**	167.1, C		167.2, C	
**12**	n/o		125.5, C	
**14**	127.4, CH	7.18 (s)	127.4, CH	7.18 (s)
**15**	n/o		121.4, C	
**16a**	19.4, CH_2	2.89 (o/l)	19.5, CH_2	2.94 (m)
**16b**		2.85 (o/l)		
**17a**	45.3, CH_2	3.92 (ddd, 3.4, 6.9, 14.2)	45.4, CH_2	3.92 (m)
**17b**		3.80 (ddd, 7.0, 13.3, 13.7)		
**19**	156.3, C		156.4, C	
**20**	103.2, C		105.6, C	
**21**	n/o		124.7, C	

Table S4. NMR data for Discorhabdophenone A (**4**) (600 (^{1}H) and 150 (^{13}C) MHz, $(CD_3)_2SO$).

pos	δ_C, type	δ_H (J in Hz)	gCOSY	gHMBC	Key ROESY
**1**	136.8, CH	6.13, s		2, 5, 6, 20	5a, 7b
**2**	132.3, C				
**3**	67.5, CH	4.59, br s	4a, 4b, OH-3		4a, 4b, 5a
**4a**	29.1, CH_2	2.06, o/l*	3, 4b, 5a, 5b	2, 3, 6	3
**4b**		1.90, o/l*	3, 4a, 5a, 5b	1, 3, 6	3
**5a**	30.6, CH_2	2.10, o/l*	4a, 4b, 5b	20	1, 3, 7b, 8b
**5b**		1.90, o/l*	4a, 4b, 5a		
**6**	38.2, C				
**7a**	30.6, CH_2	2.06, o/l*	7b, 8a, 8b	6	
**7b**		1.63, td (3.1, 12.6)	7a, 8a, 8b	1, 5, 6, 8	8a, 8b
**8a**	37.0, CH_2	3.55, dt (3.1, 13.3)	7a, 7b, 8b	6	7b, 8b, 5a
**8b**		3.32, o/l*	8a, 7a, 7b	5	8a, 7b
**10**	145.1, C				
**11**	168.8, C				
**12**	132.7, C				
**14**	150.5, C				
**15**	121.6, C				
**16**	111.5, CH	7.84, d (5.7)		15, 17	
**17**	129.4, CH	7.91, d (5.7)	18	16, 19, 21	
**18**		11.26, br s	17		
**19**	138.8, C				
**20**	100.7, C				
**21**	131.6, C				
**22**	188.6, C				
**23**	138.4, C				
**24**	130.3, 2 X CH	8.37, d (5.6)	25	22, 24, 26	
**25**	127.9, 2 X CH	7.56, t (7.5)	24, 26	23, 24, 25	
**26**	131.8, CH	7.63, t (6.9)	25	24	
**OH-3**		5.35, br s	3		

* Overlapping ^{1}H NMR signals, 2D assignments based on proximity likelihood.

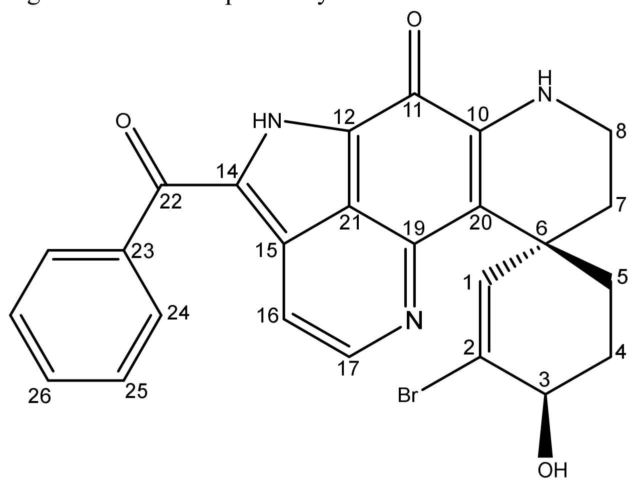

Figure S17. Structure of **4**.

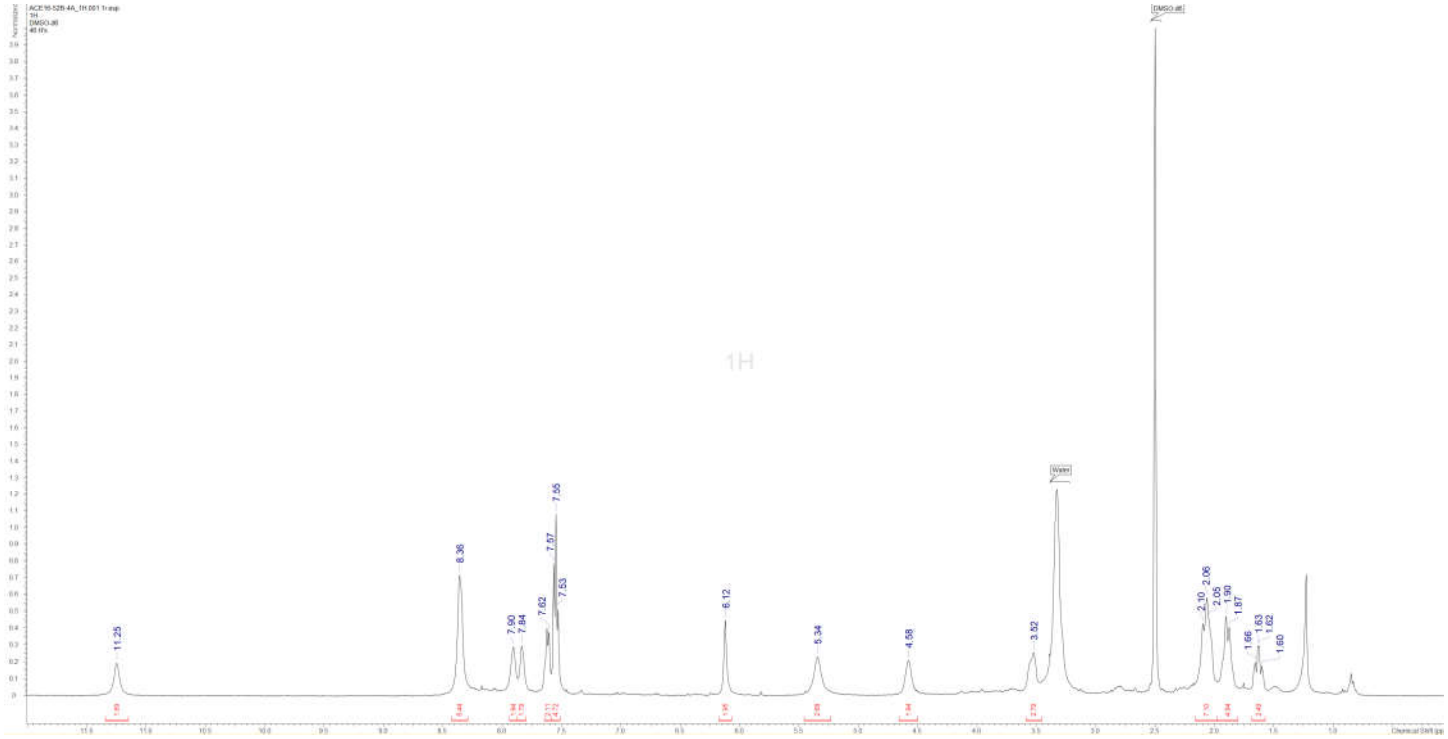

Figure S18. Full scale (0.0-12 ppm) ^{1}H NMR spectrum (600 MHz, $(CD_3)_2SO$) of **4**.

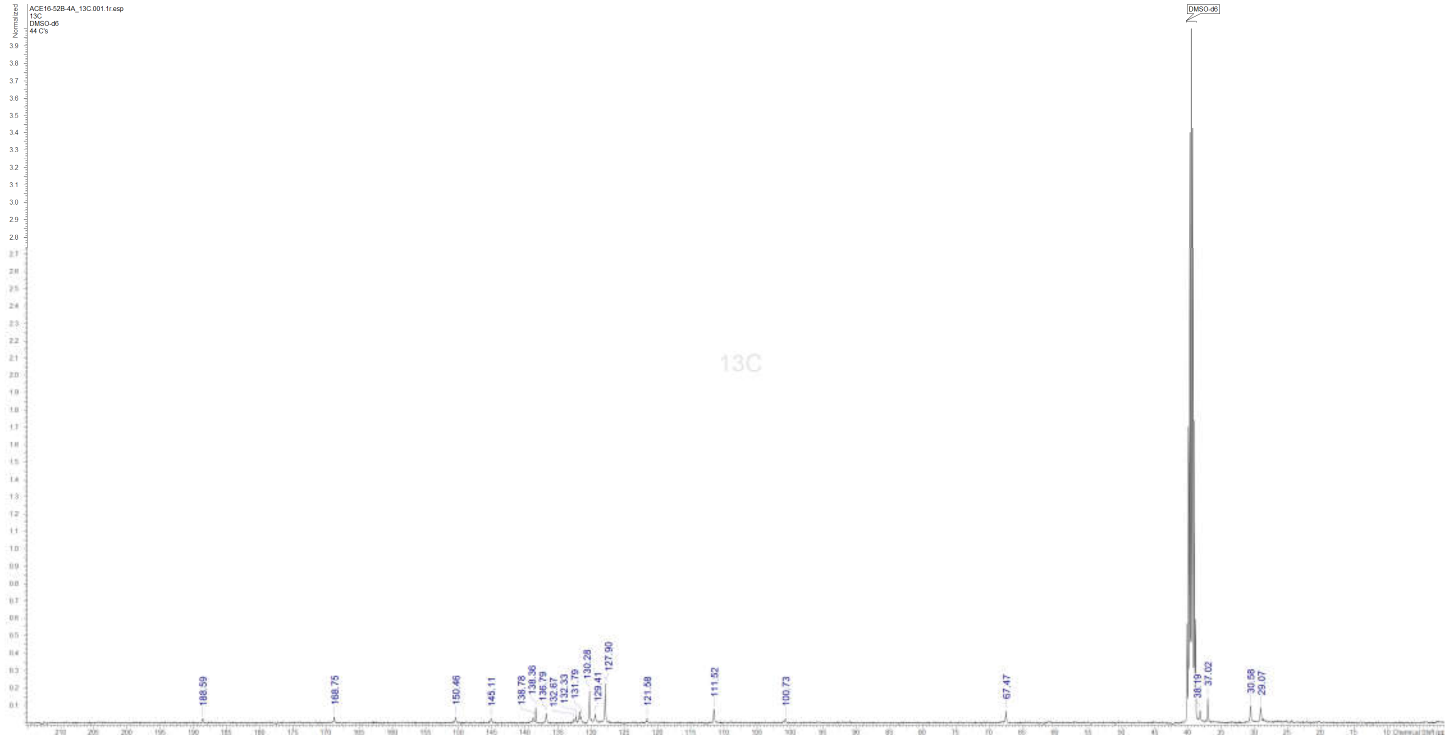

Figure S19. Full spectrum (0-215 ppm) BB ^{13}C NMR spectrum (150 MHz, $(CD_3)_2SO$) of **4**.

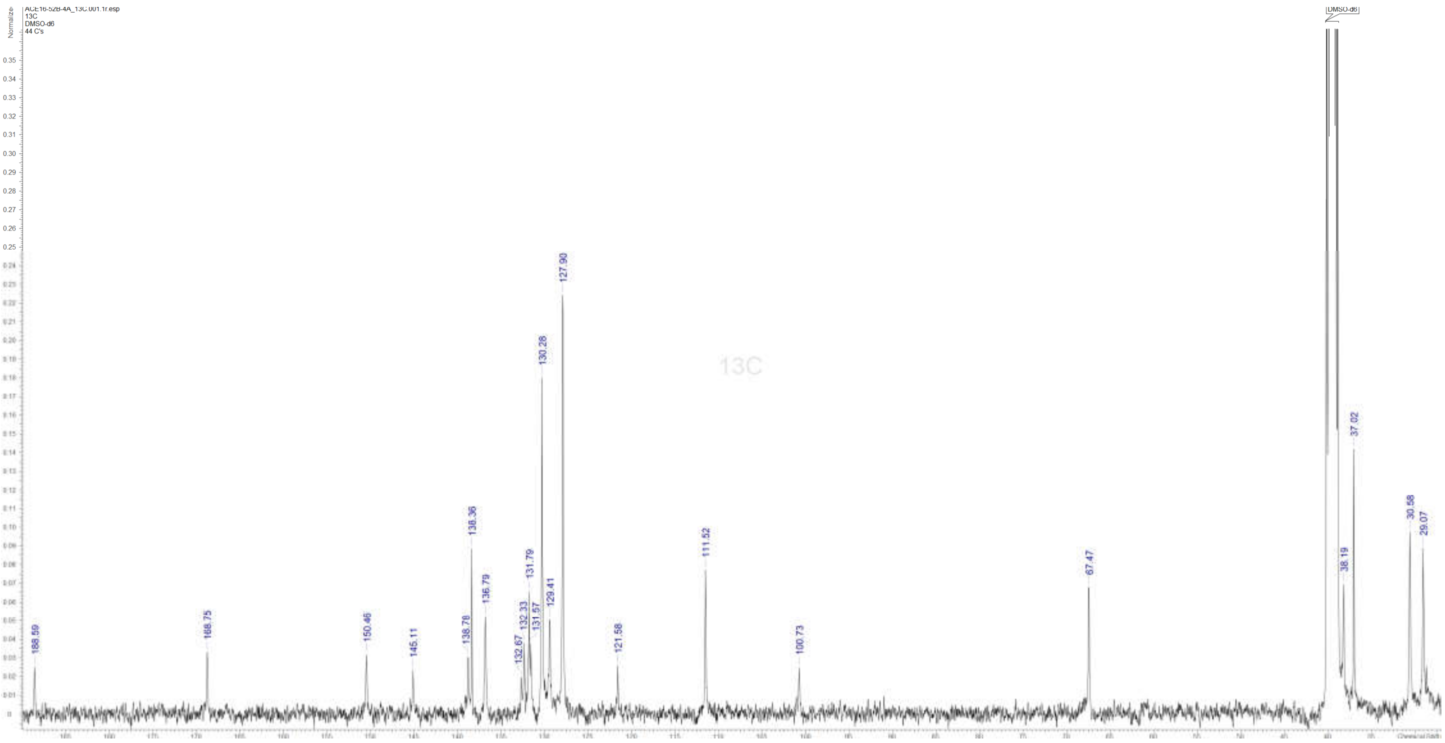

Figure S20. Expanded (28-190 ppm) BB ^{13}C NMR spectrum (150 MHz, $(CD_3)_2SO$) of **4**.

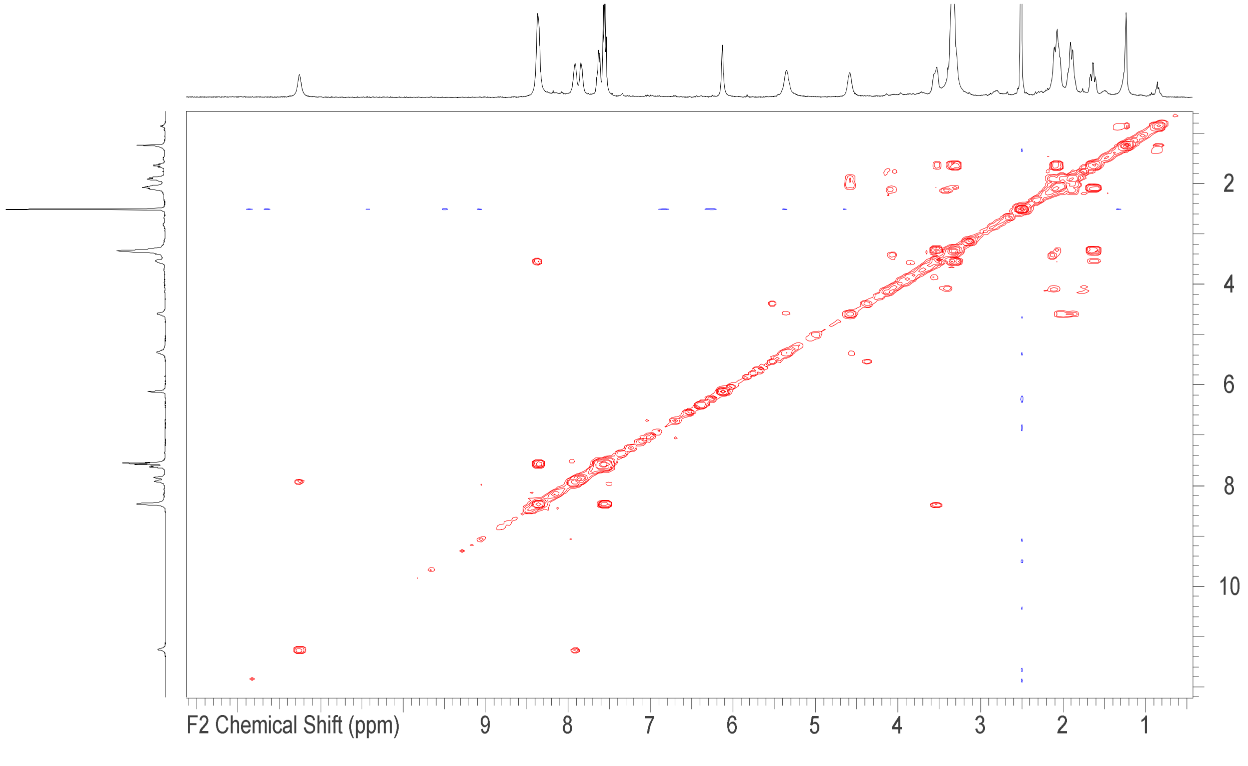

Figure S21. COSY NMR spectrum (600 MHz, $(CD_3)_2SO$) of **4**.

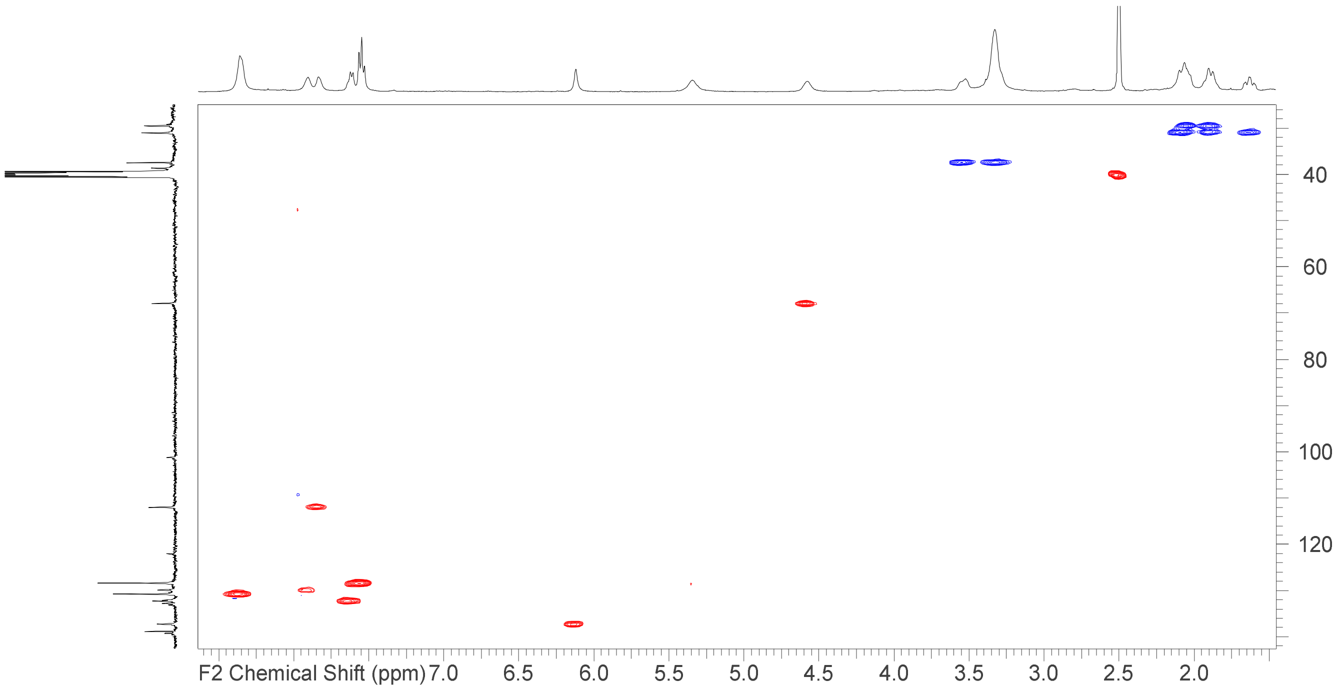

Figure S22. HSQC-DEPT NMR spectrum (600 MHz, $(CD_3)_2SO$) of **4**.

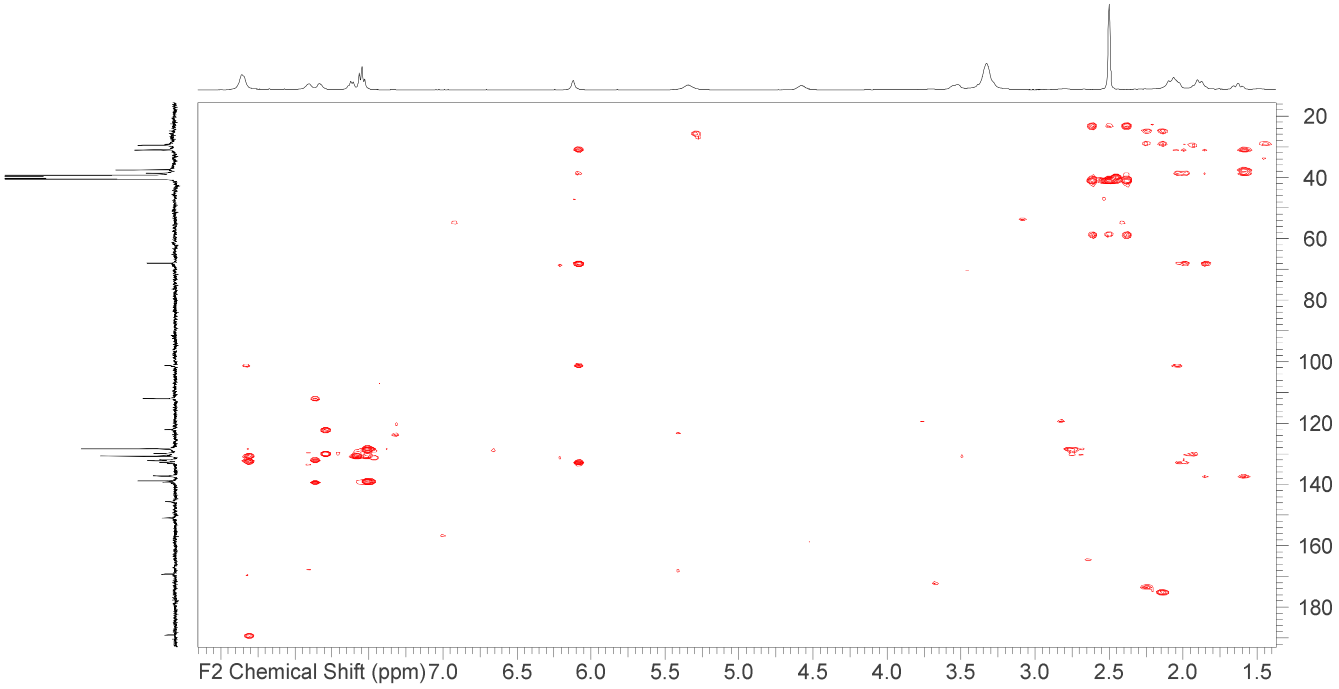

Figure S23. HMBC NMR spectrum (600 MHz, $(CD_3)_2SO$) of **4**.

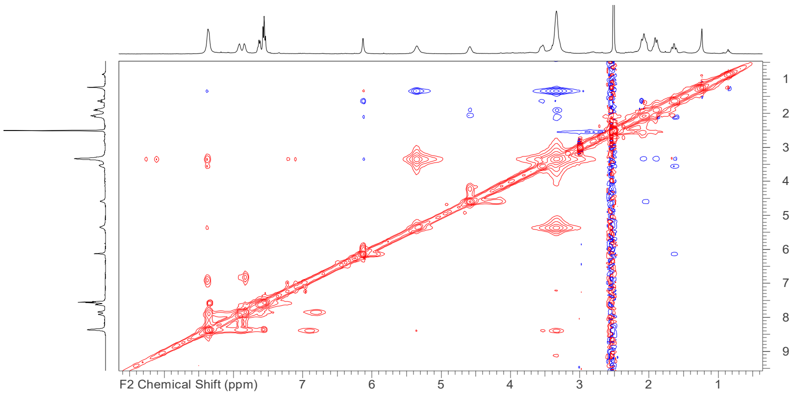

Figure S24. NOESY NMR spectrum (600 MHz, $(CD_3)_2SO$) of **4**.

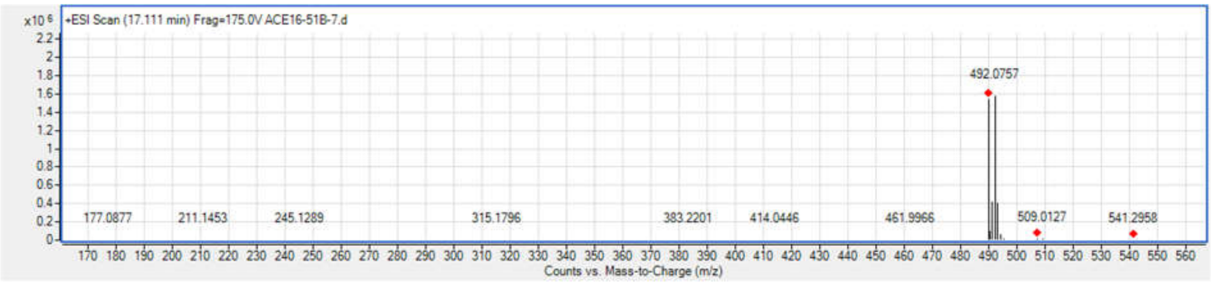

Figure S25. HRESIMS analysis of **4**.

Table S5. NMR data for discorhabdophenone B (**5**) (600 (^{1}H) and 150 (^{13}C) MHz, $(CD_3)_2SO$).

pos	δ_C, type	δ_H (*J* in Hz)	gCOSY	gHMBC	Key NOESY
**1**	67.2, CH	4.84, dd (1.2, 1.2)	2, OH-1		7b, OH-1
**2**	65.7, CH	5.02, d (1.2)	1	1, 3, 6, 17	17
**3**	184.5, C				
**4**	109.9, CH	5.96, s		2, 5, 6, 20	
**5**	171.3, C	7.94			
**6**	47.5, C				
**7a**	36.0, CH_2	2.86, dd (10.9, 2.4)	7b, 8	6, 8, 20	7b
**7b**		2.61, dd (11.2, <1)	7a	5, 6, 8, 20	7a
**8**	63.4, CH	5.70, dd (2.7, 2.7)	7a, 9	5, 10	7a, 7b
**9**		9.30, s	8	8	
**10**	145.5, C				
**11**	168.7, C				
**12**	132.3, C				
**14**	130.8, C				
**15**	121.2, C				
**16**	115.1, CH	7.94, (6.4)		17, 19	
**17**	131.5, CH	8.05, (6.4)		2, 12, 16	2
**19**	146.0, C				
**20**	102.2, C				
**21**	132.7, C				
**22**	186.2, C				
**23**	129.2, C				
**24**	133.1, 2 X CH	6.90, d (8.0)	24	22, 25, 26	
**25**	114.8, 2 X CH	8.42, d (8.2)	25	23, 24, 26	
**26**	161.5, C				
**OH-3**		6.75, br s	3		3
**OH-26**		10.28, br s			

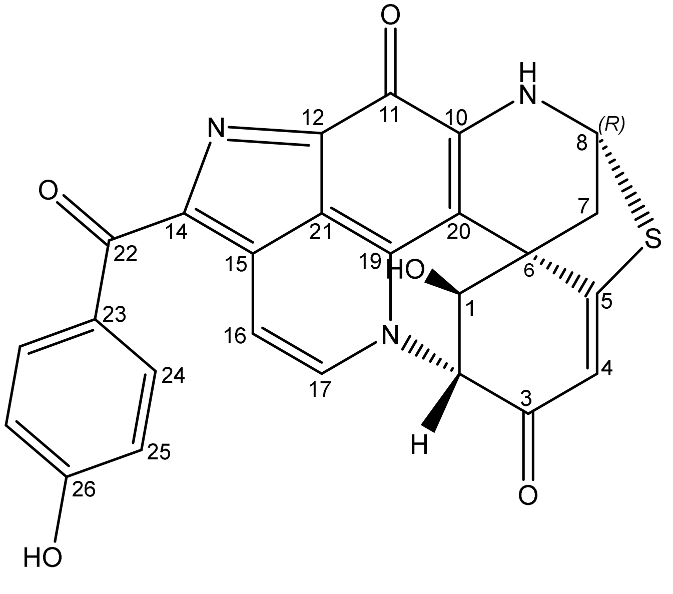

Figure S26. Chemical structure of **5**.

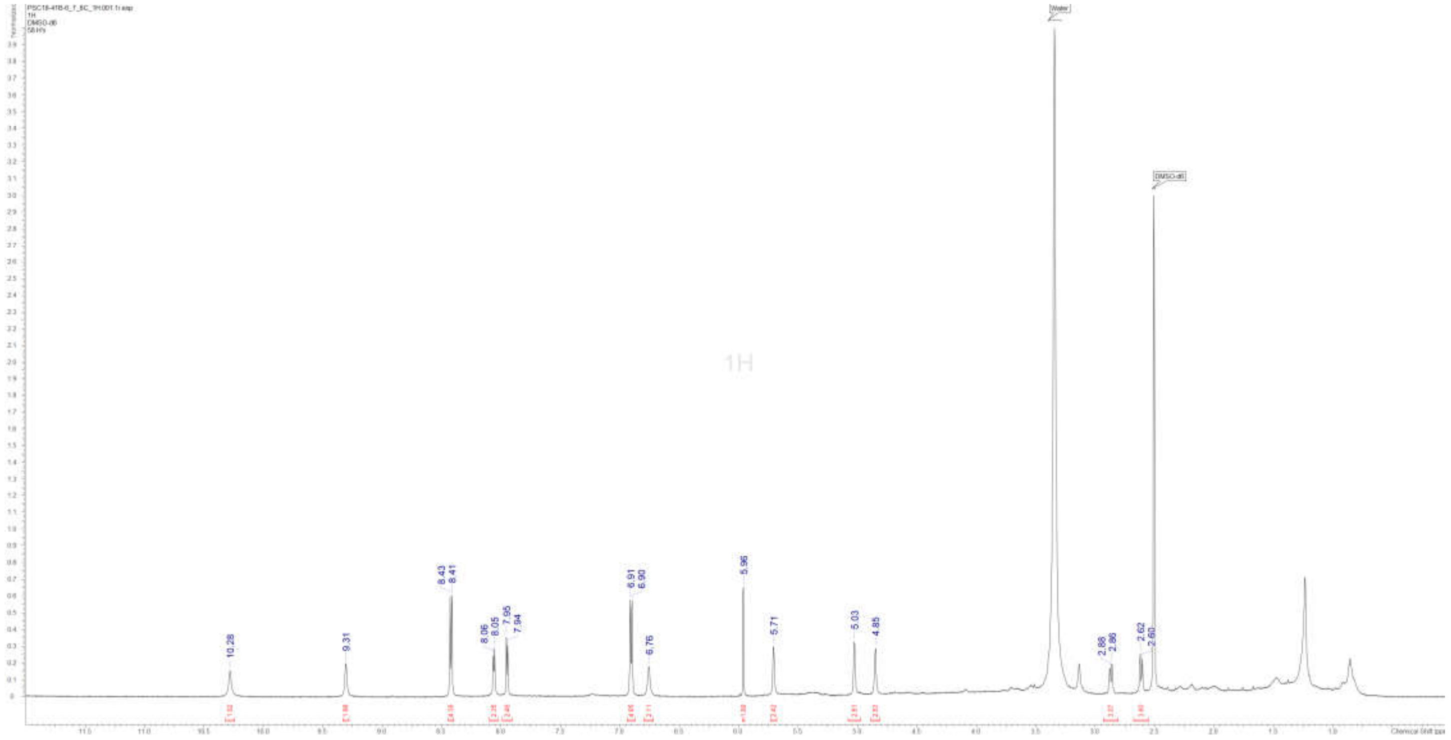

Figure S27. Full scale (0.0-12.0 ppm) ^{1}H NMR spectrum (600 MHz, $(CD_3)_2SO$) of **5**.

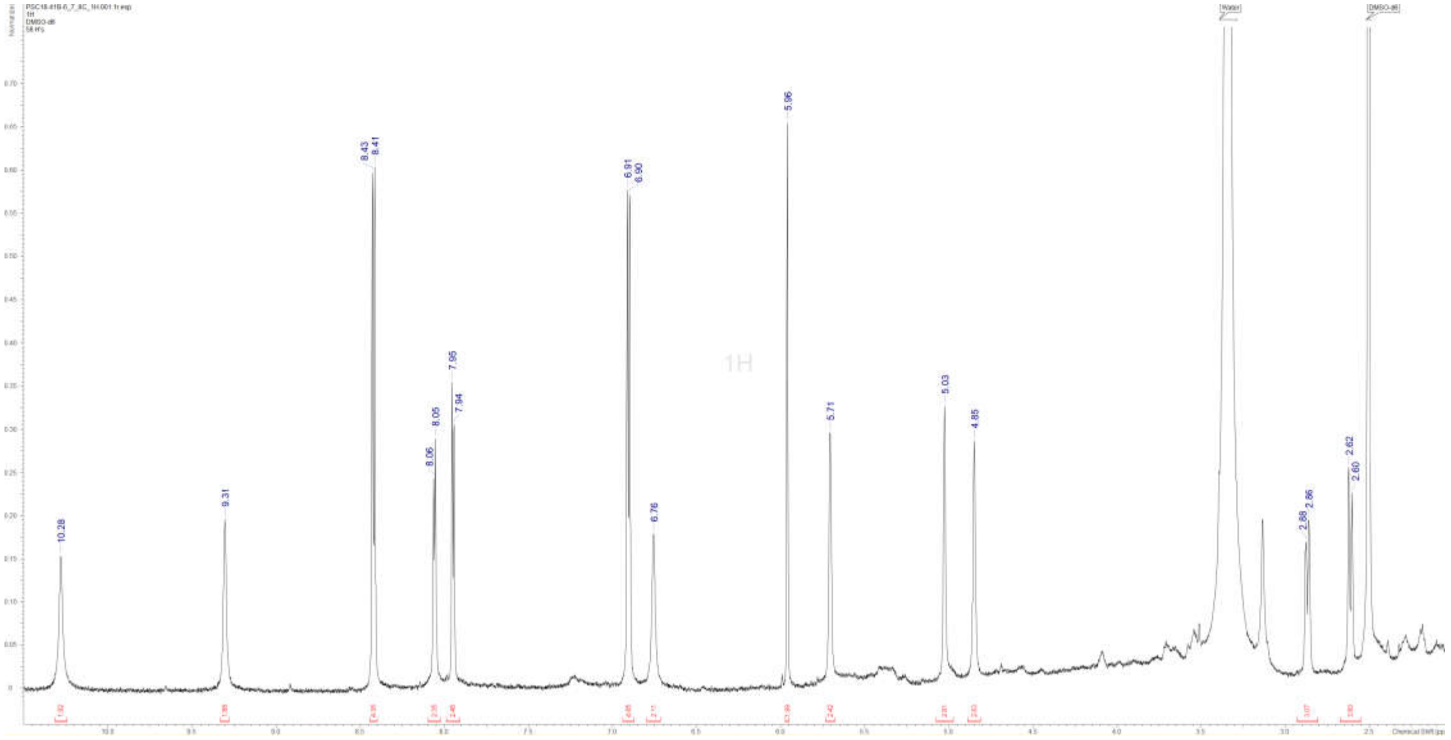

Figure S28. Expanded (2.0-10.5 ppm) ^{1}H NMR spectrum (600 MHz, $(CD_3)_2SO$) of **5**.

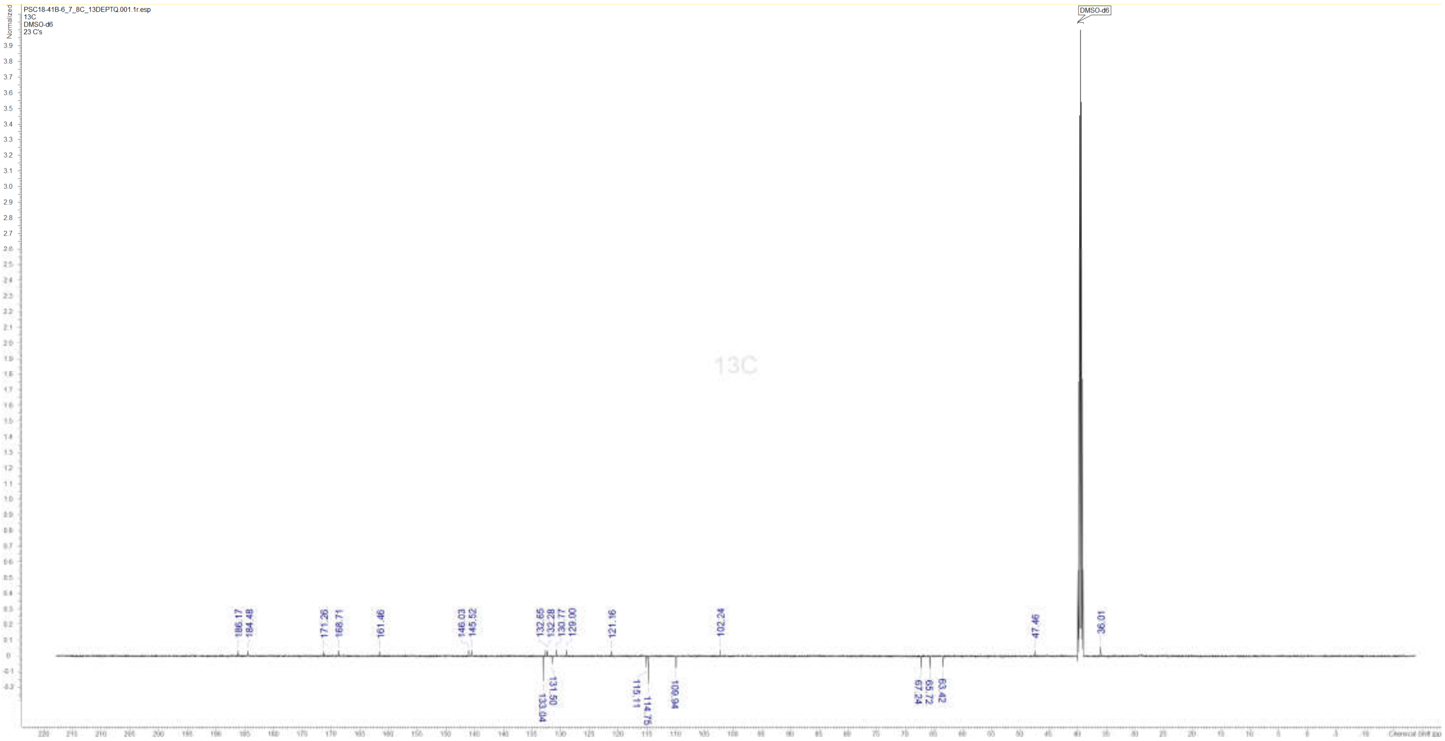

Figure S29. Full scale (-20.0-220.0 ppm) DEPTQ135 NMR spectrum (150 MHz, $(CD_3)_2SO$) of **5**.

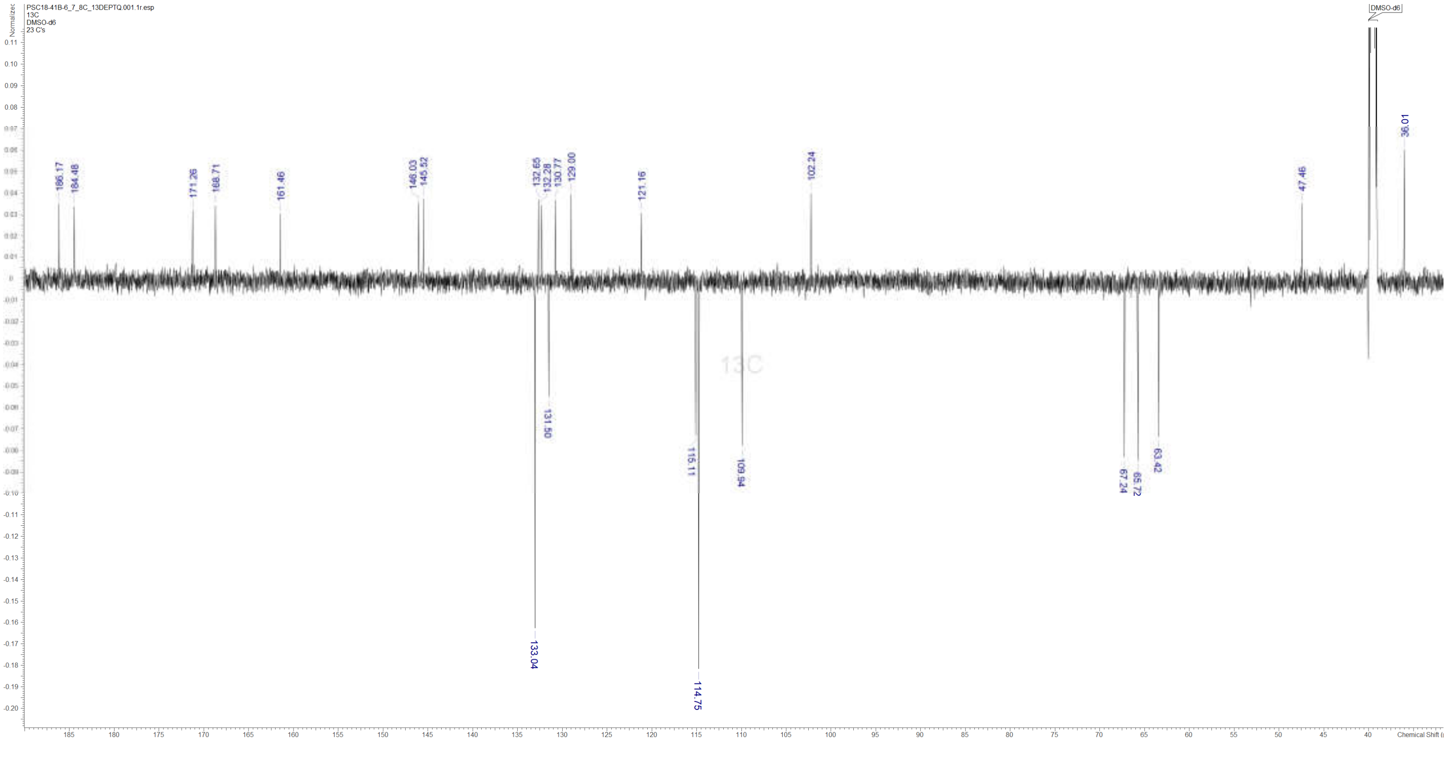

Figure S30. Expanded (30.0-190.0 ppm) DEPTQ135 NMR spectrum (150 MHz, $(CD_3)_2SO$) of **5**.

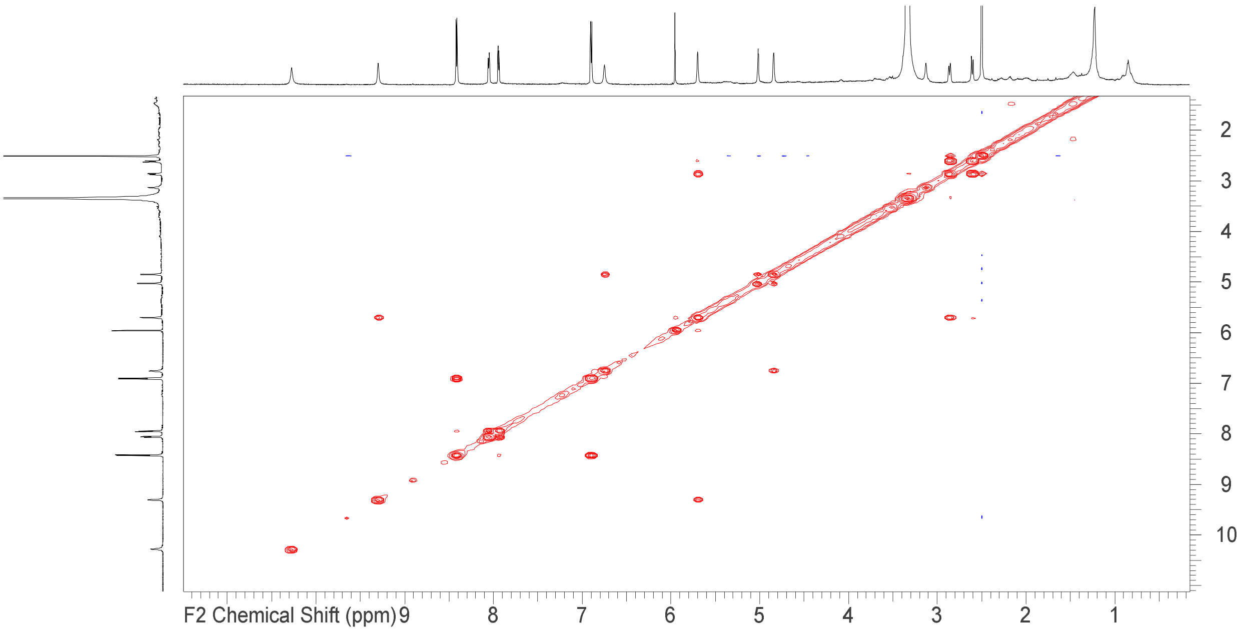

Figure S31. COSY NMR spectrum (600 MHz, $(CD_3)_2SO$) of **5**.

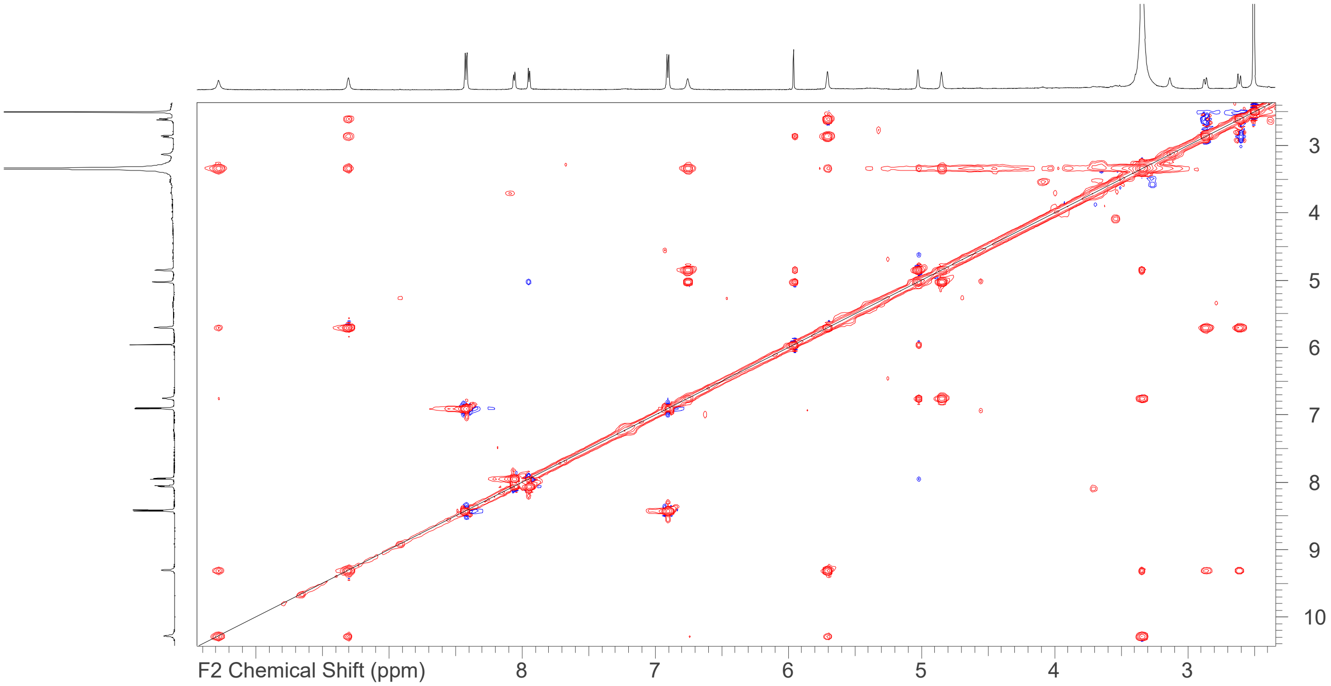

Figure S32. TOCSY NMR spectrum (600 MHz, $(CD_3)_2SO$) of **5**.

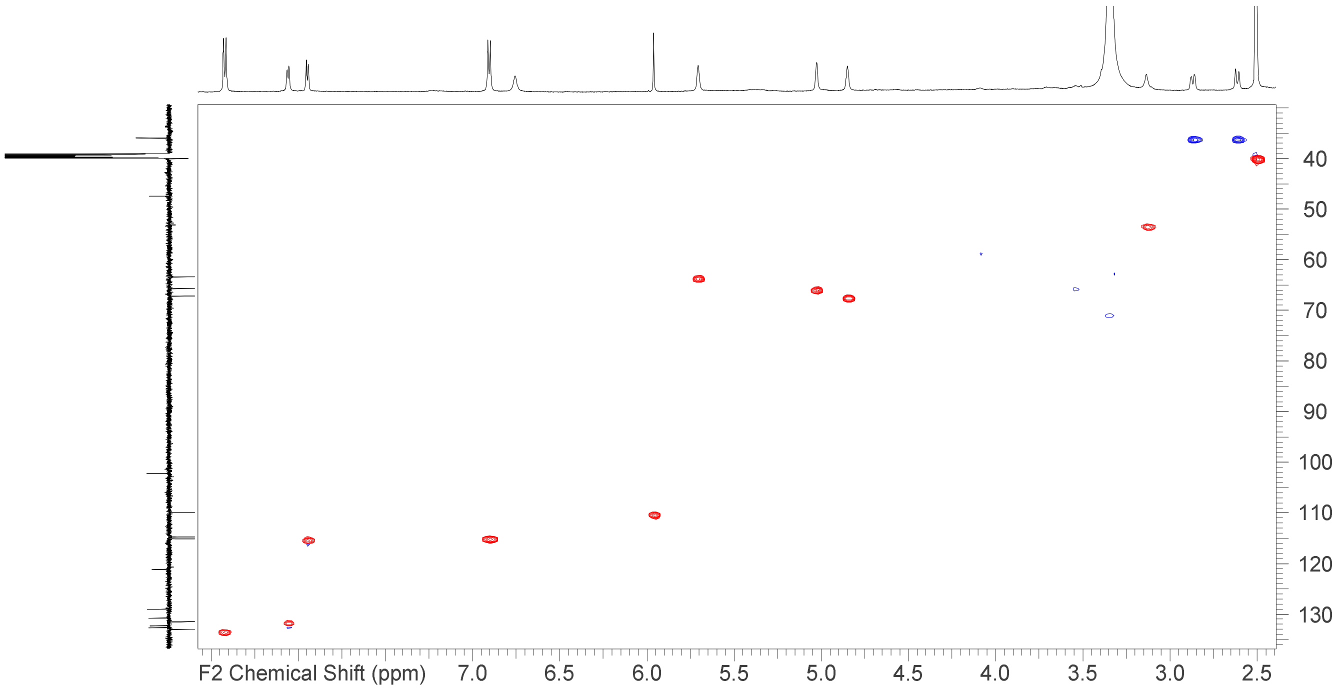

Figure S33. HSQC-DEPT NMR spectrum (600 MHz, $(CD_3)_2SO$) of **5** with the DEPTQ spectrum on F1 axis.

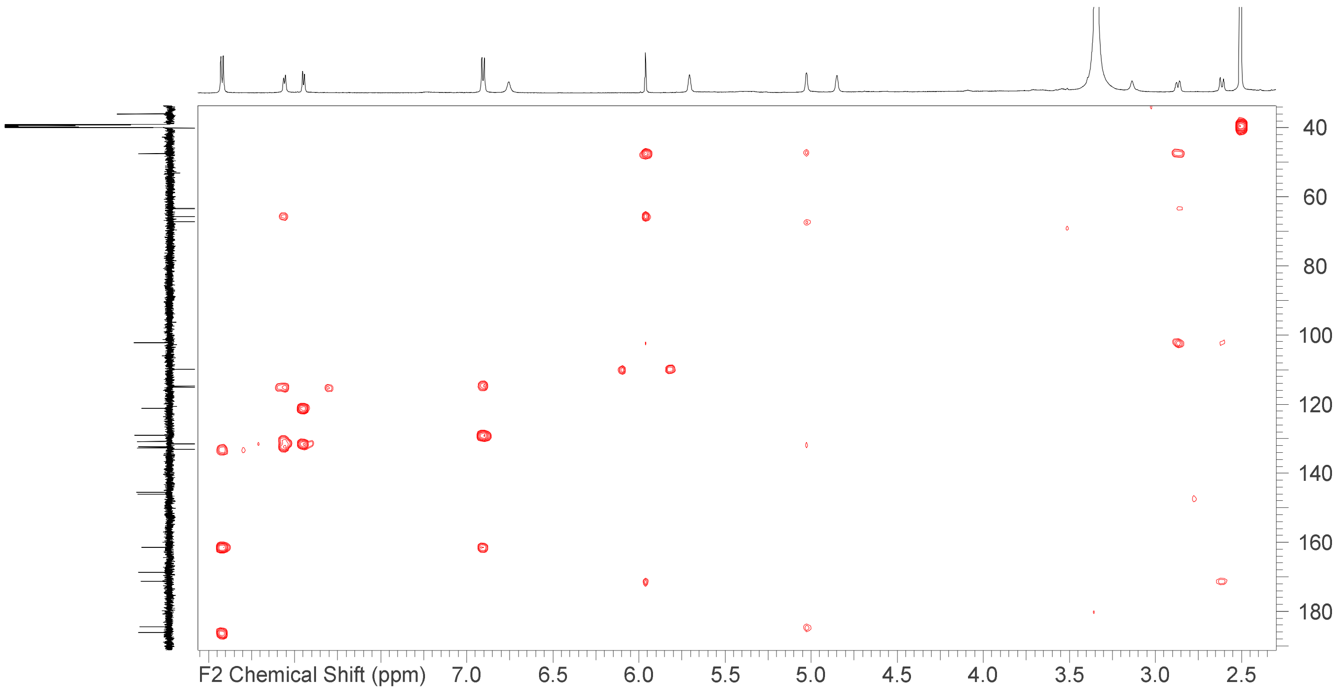

Figure S34. HMBC NMR spectrum (600 MHz, $(CD_3)_2SO$) of **5** with DEPTQ spectrum on F1 axis.

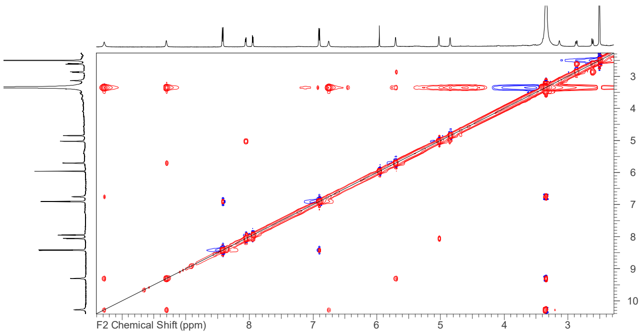

Figure S35. NOESY NMR spectrum (600 MHz, $(CD_3)_2SO$) of **5**.

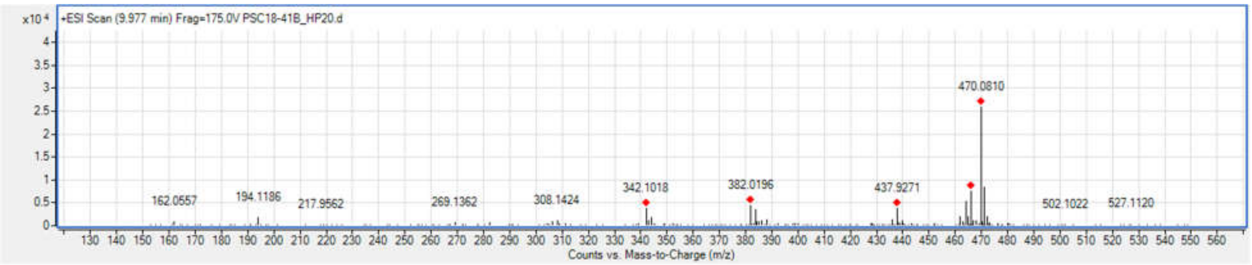

Figure S36. HRESIMS analysis of **5**.

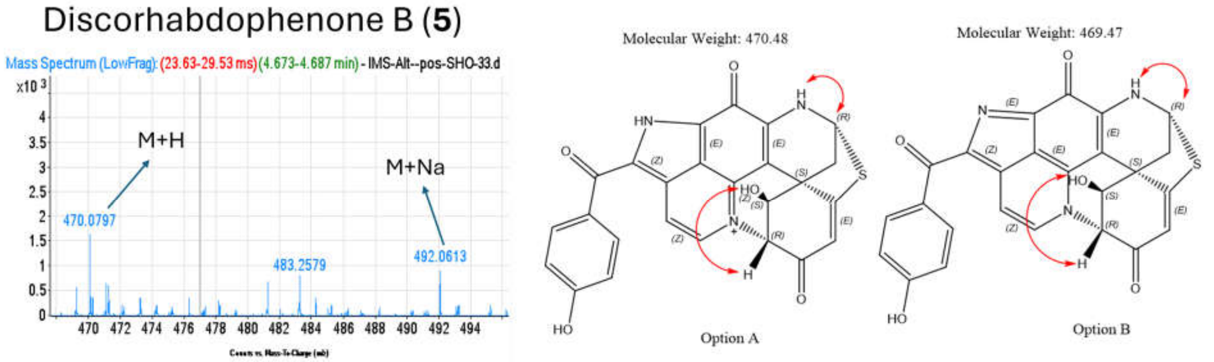

Data type	Observed	Option A	Option B
No. of Exchangeable Protons	3	4	3
M+H	470	471	470
M+Na	492	493	492

Figure S37. Analysis of the protonation state of **5**.

Table S6. NMR data for discorhabdophenone C (**6**) (600 (^{1}H) and 150 (^{13}C) MHz, $(CD_3)_2SO$).

pos	δ_C, type	δ_H (*J* in Hz)	gCOSY	gHMBC	Key NOESY
**1**	67.2, CH	4.85, dd (1.8, 1.8)	2, OH-1	3, 5	2, 7b, OH-1
**2**	65.7, CH	5.04, d (1.9)	1	3, 3, 4	3, 4, 17, OH-1
**3**	184.5, C				
**4**	110.0, CH	5.97, s		2, 5, 6	
**5**	171.2, C				
**6**	47.5, C				
**7a**	36.0, CH_2	2.86, dd (11.6, 2.8)	7b, 8	6, 20	OH-3
**7b**		2.61, dd (11.6, <1)	7a	5, 20	3
**8**	63.4, CH	5.71, dd (2.2, 1.0)	7a		
**9**		9.34, s	8		
**10**	145.2, C				
**11**	169.1, C				
**12**	133.2, C				
**14**	130.7, C				
**15**	121.3, C				
**16**	114.9, CH	7.97, d (6.7)		15, 17	
**17**	131.9, CH	8.10, d (6.5)		2, 16	2
**19**	145.4, C				
**20**	102.4, C				
**21**	132.5, C				
**22**	188.2, C				
**23**	138.0, C				
**24**	130.4, 2 X CH	8.35, d (7.8)	25	22, 24, 26	
**25**	128.0, 2 X CH	7.55, t (7.6)	24, 26	23, 25	
**26**	132.1, CH	7.63, t (7.4)	25	24	
**OH-3**		6.76, br s	3	2, 3	2, 3, 7a

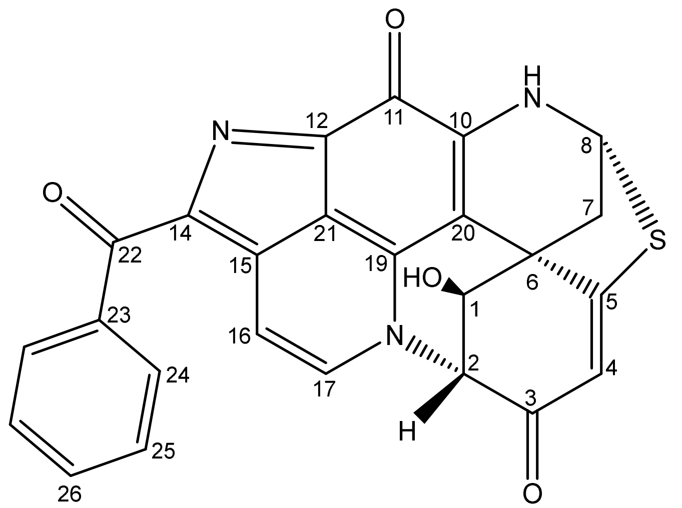

Figure S38. Chemical structure of **6**.

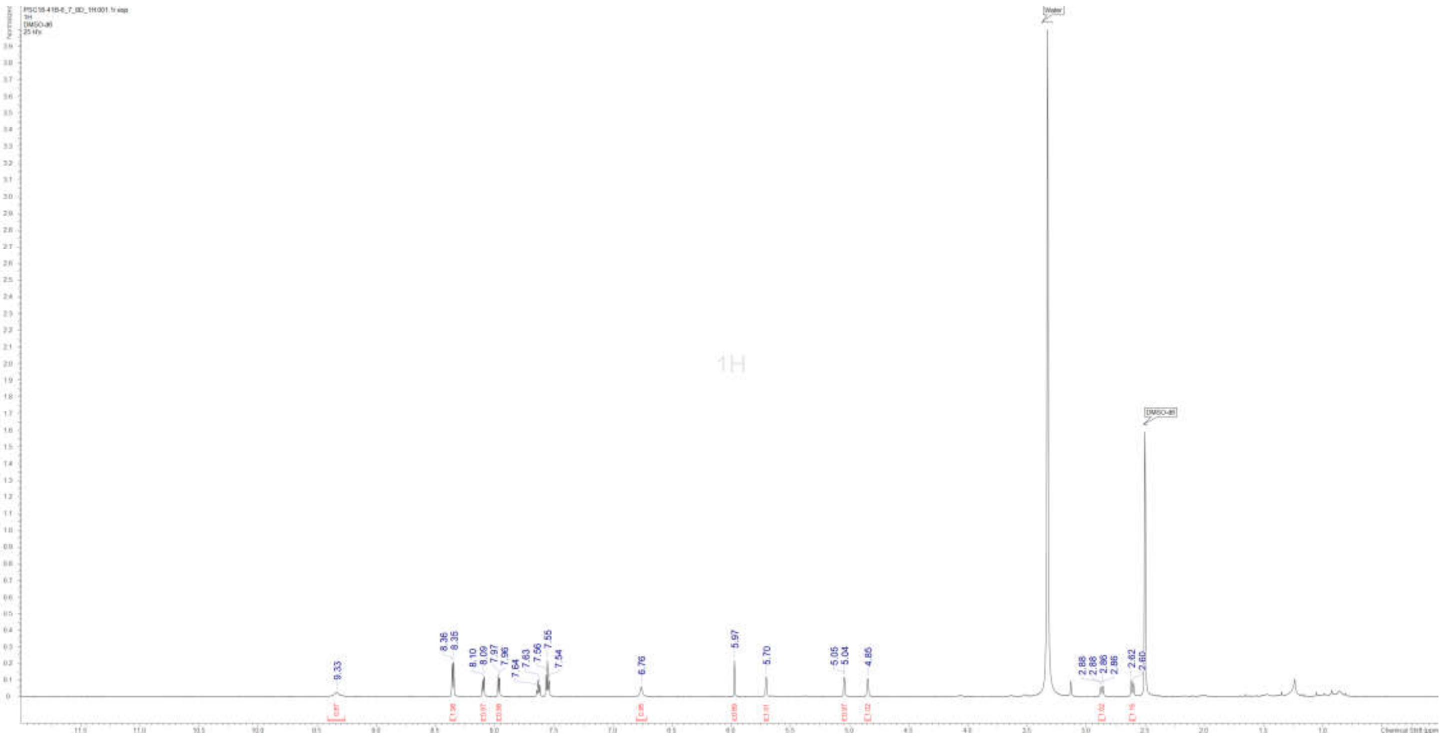

Figure S39. Full scale (0.0-12.0 ppm) ^{1}H NMR spectrum (600 MHz, $(CD_3)_2SO$) of **6**.

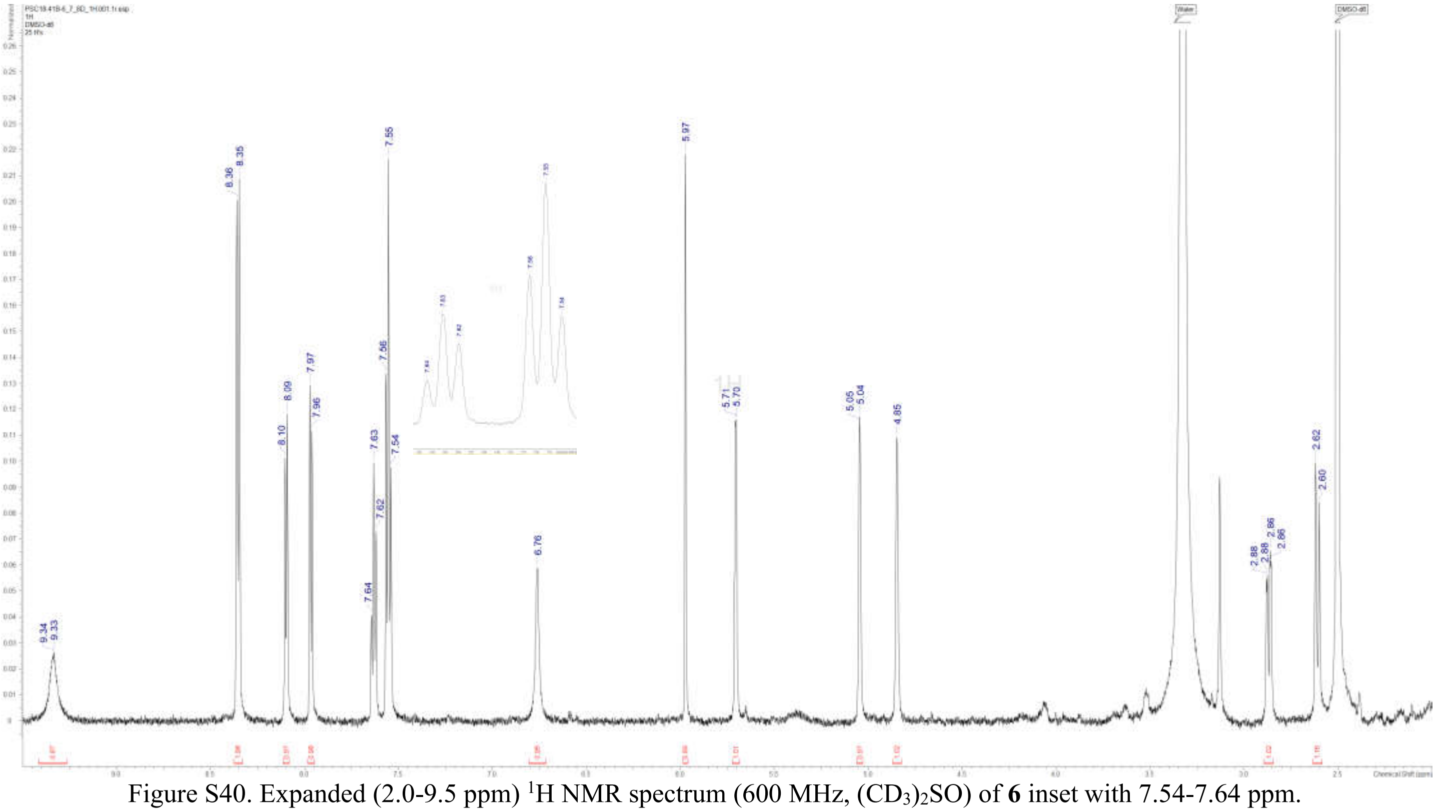

Figure S40. Expanded (2.0-9.5 ppm) ^{1}H NMR spectrum (600 MHz, $(CD_3)_2SO$) of **6** inset with 7.54-7.64 ppm.

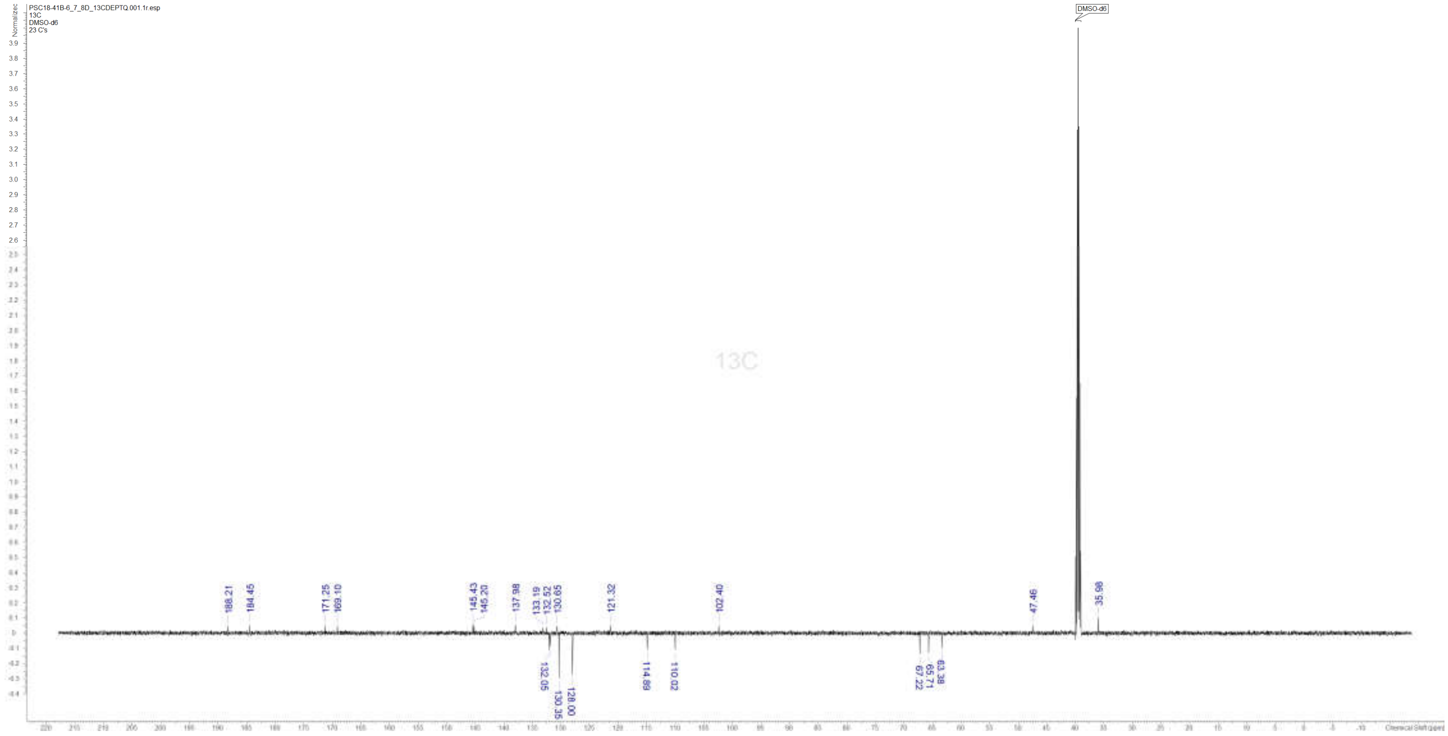

Figure S41. Full scale (-20.0-220.0 ppm) DEPTQ135 NMR spectrum (150 MHz, $(CD_3)_2SO$) of **6**.

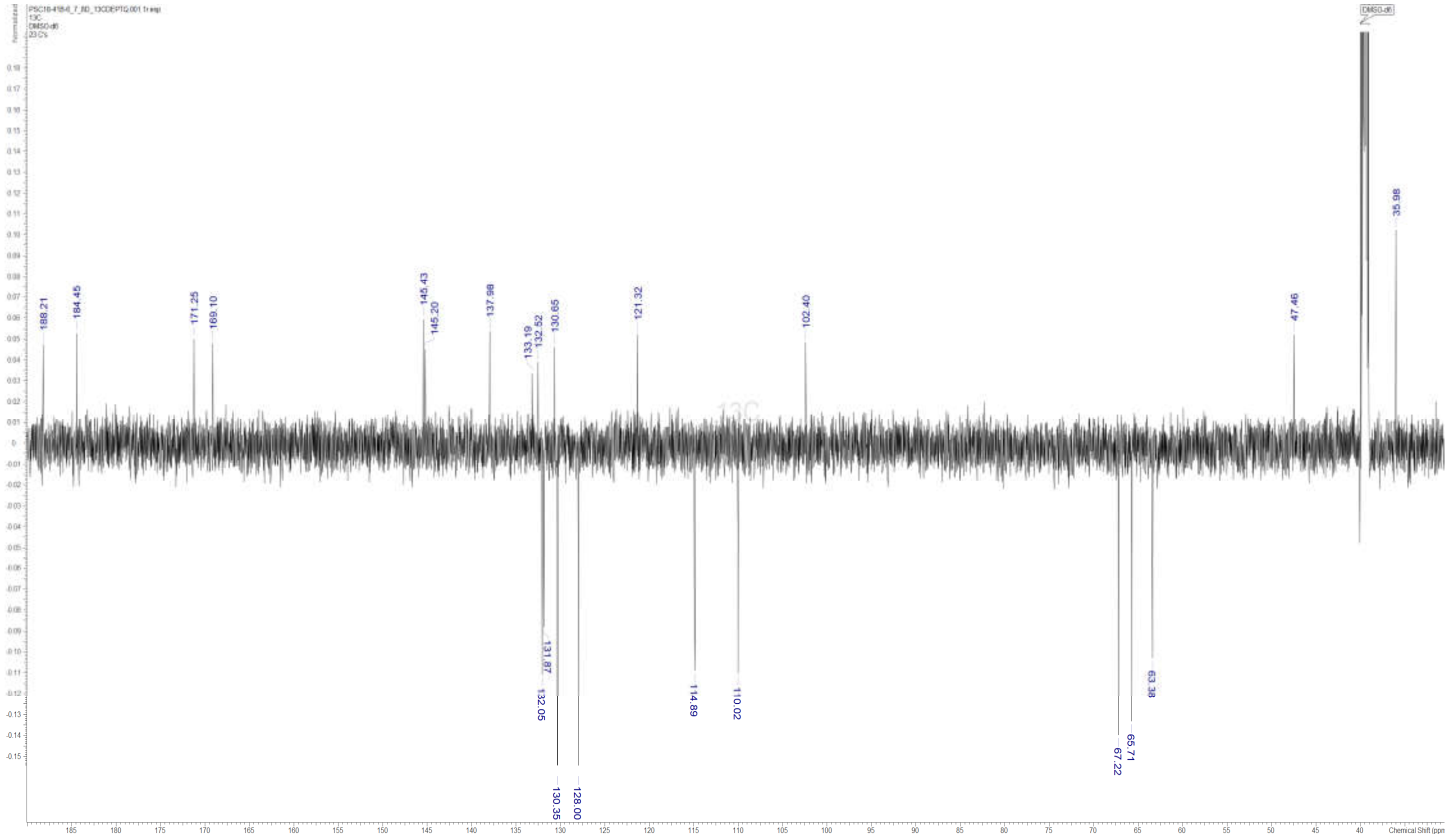

Figure S42. Expanded (30.0-190.0 ppm) DEPTQ135 NMR spectrum (150 MHz, $(CD_3)_2SO$) of **6**.

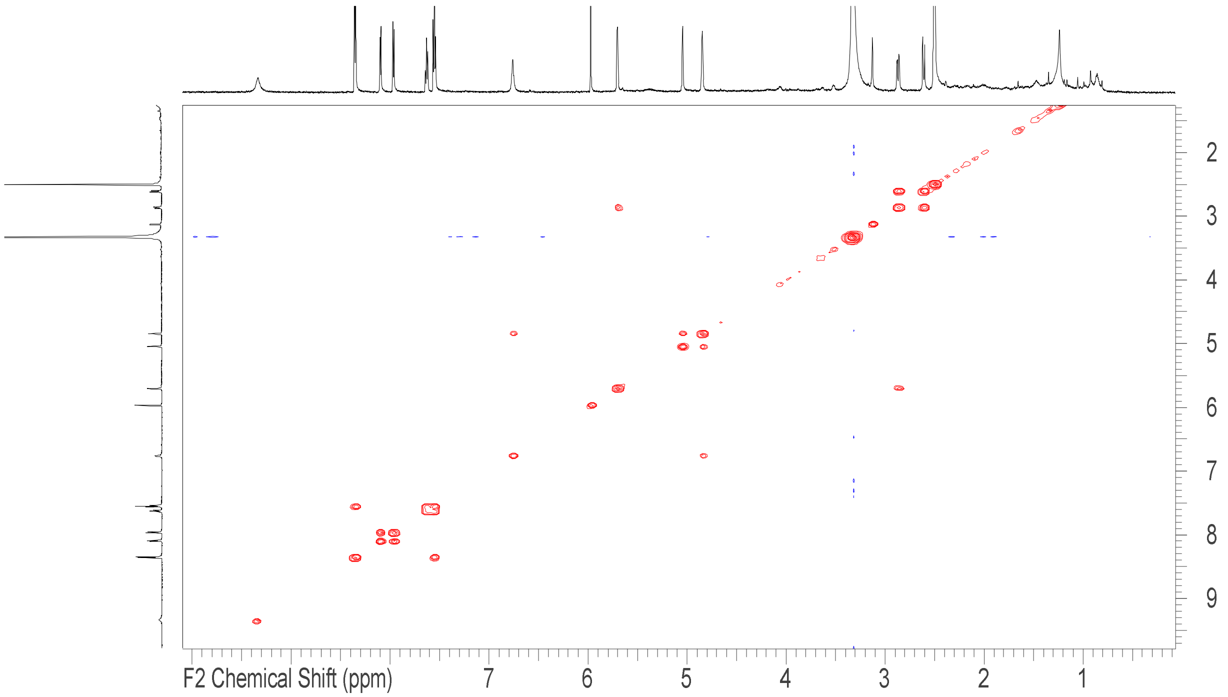

Figure S43. COSY NMR spectrum (600 MHz, $(CD_3)_2SO$) of **6**.

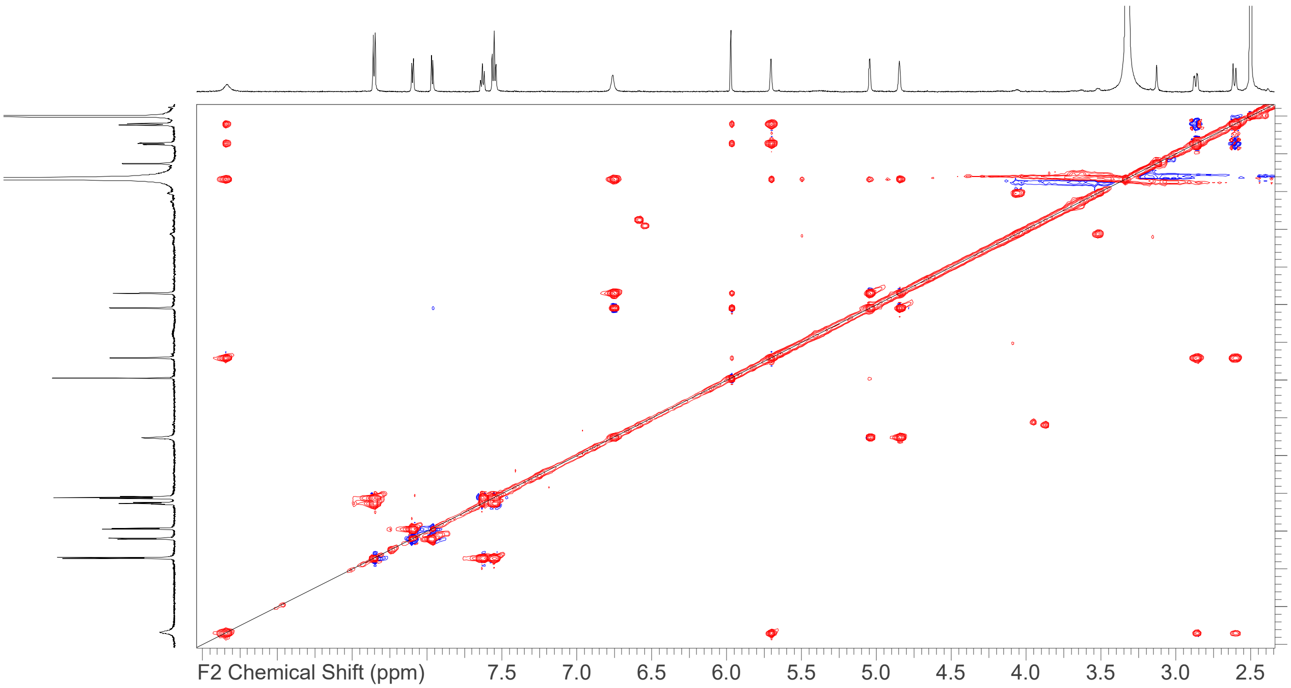

Figure S44. TOCSY NMR spectrum (600 MHz, $(CD_3)_2SO$) of **6**.

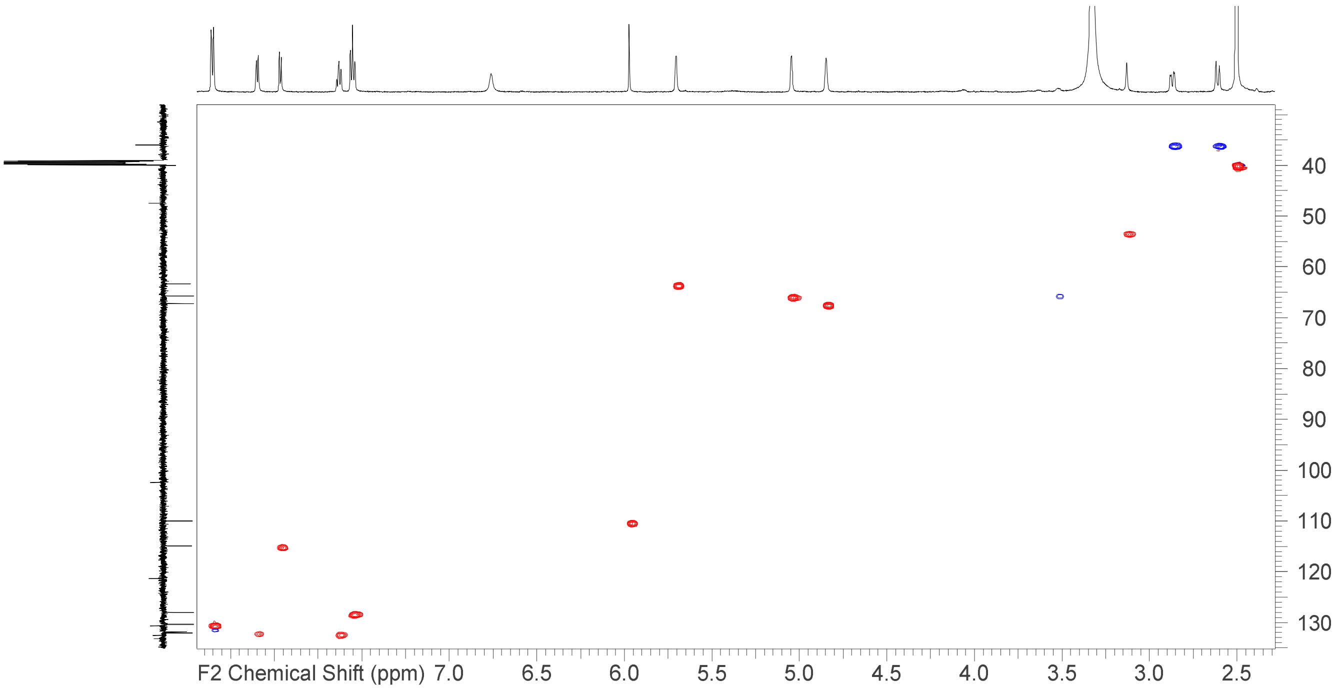

Figure S45. HSQC-DEPT NMR spectrum (600 MHz, $(CD_3)_2SO$) of **6** with DEPTQ135 on F1 axis.

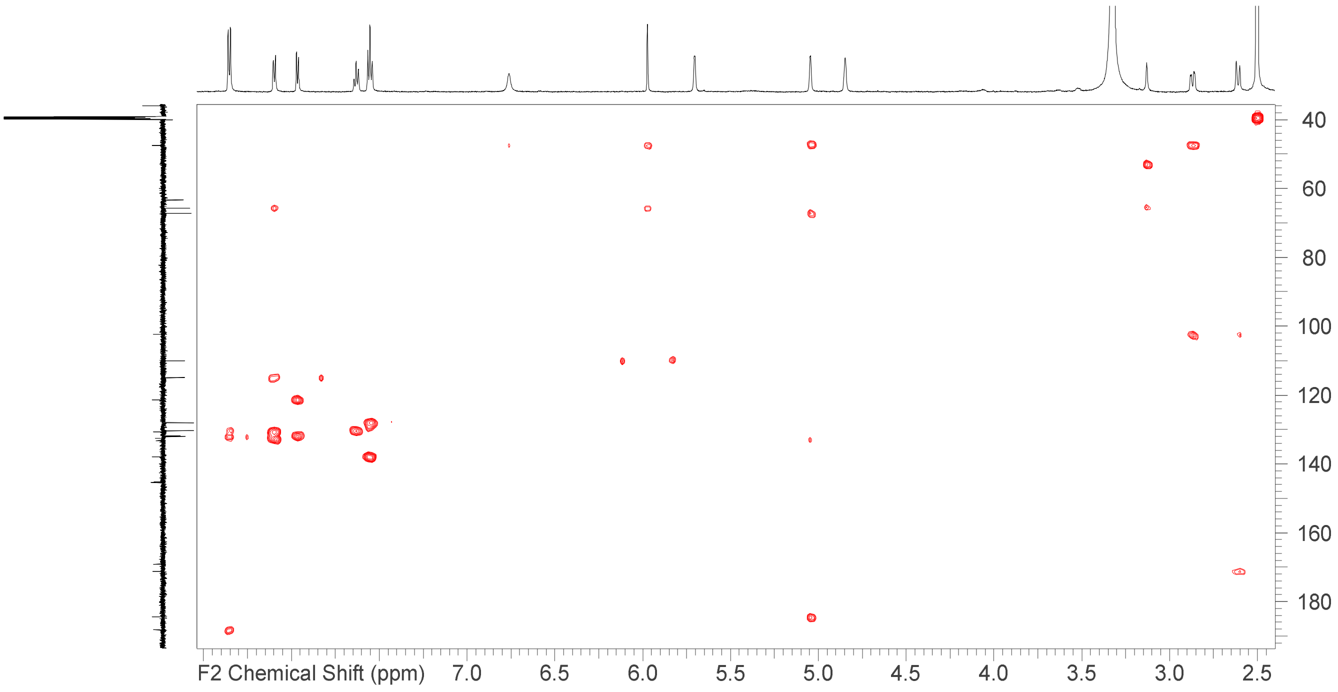

Figure S46. HMBC NMR spectrum (600 MHz, $(CD_3)_2SO$) of **6**.

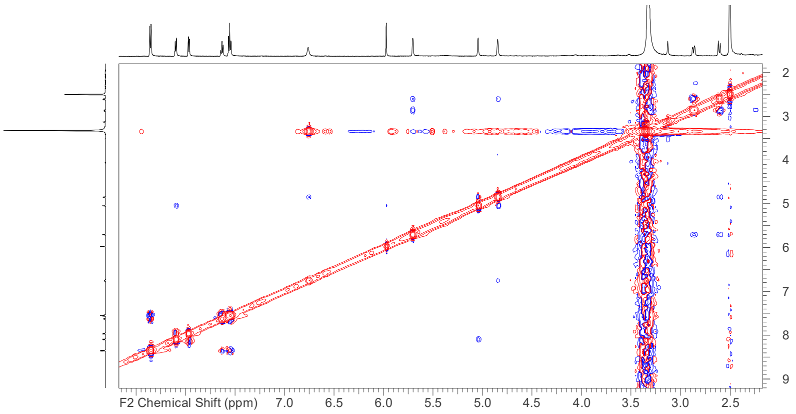

Figure S47. NOESY NMR spectrum (600 MHz, $(CD_3)_2SO$) of **6**.

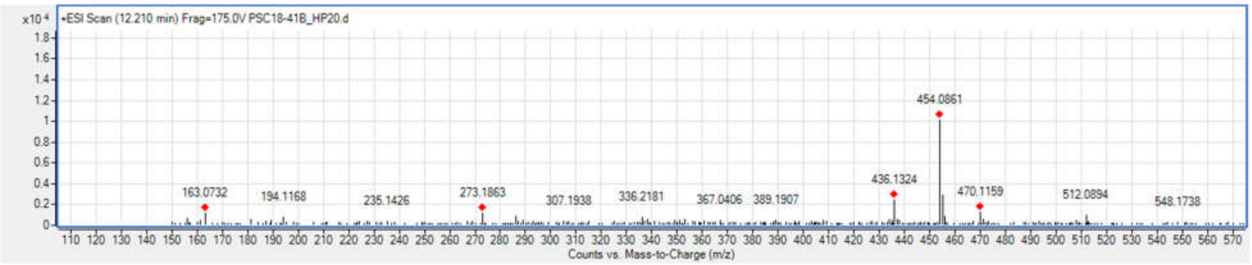

Figure S48. HRESIMS analysis of **6**.

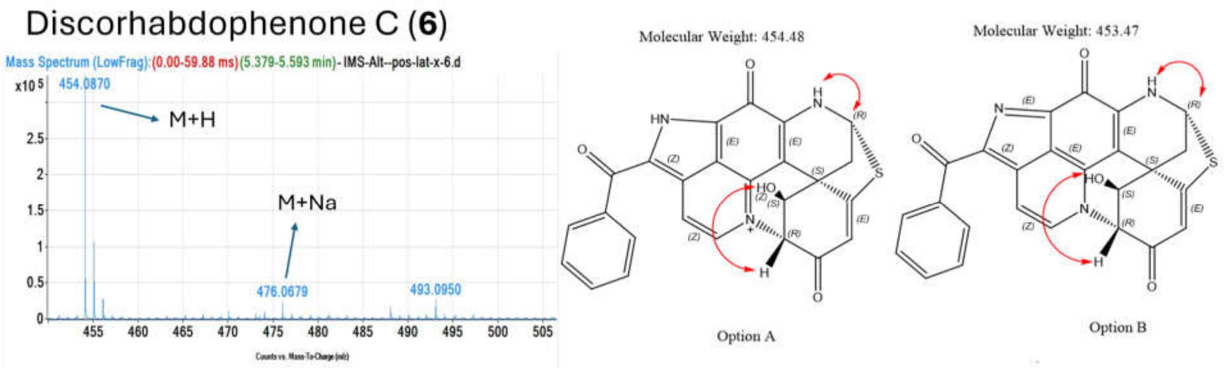

Data type	Observed	Option A	Option B
No. of Exchangeable Protons	2	3	2
M+H	454	455	454
M+Na	476	477	476

Figure S49. Analysis of the protonation state of **6**.

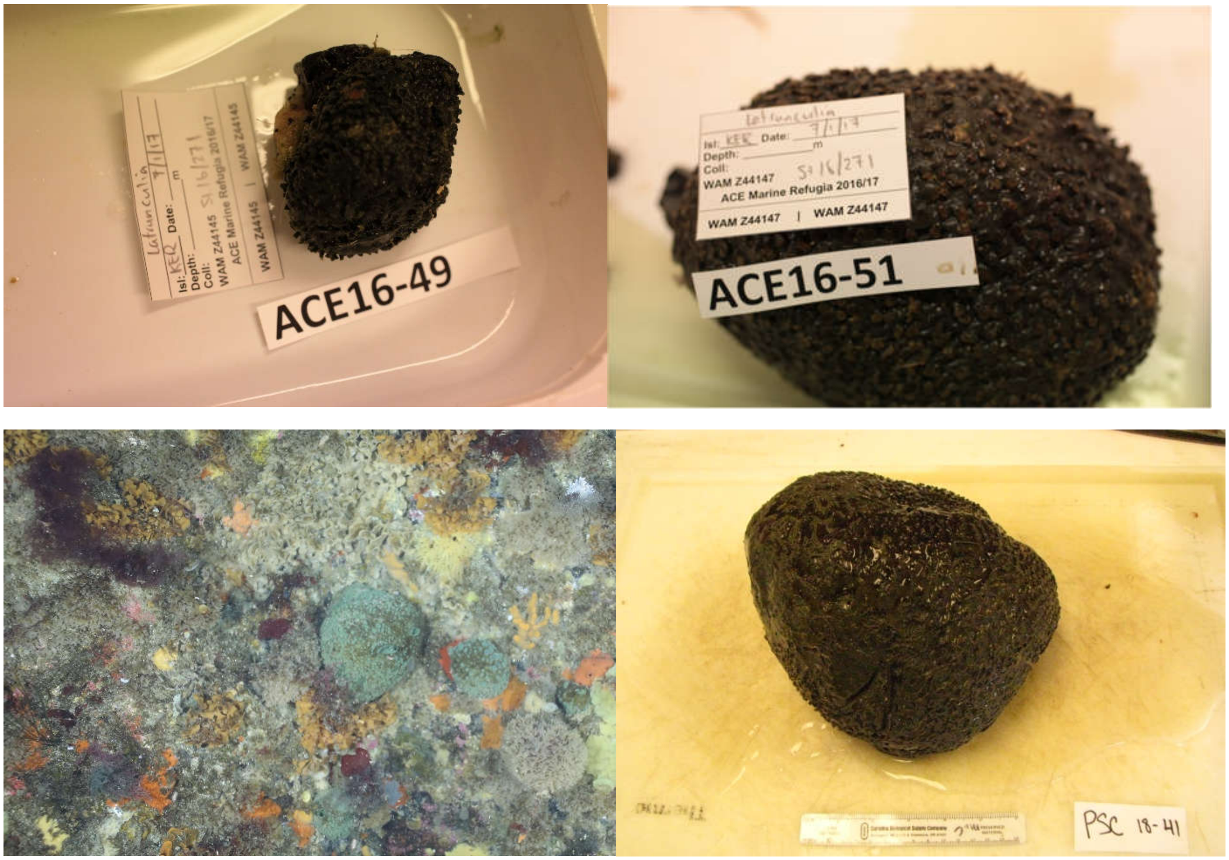

Figure S50. Photo documentation of specimens. Top: Voucher photos of ACE16 specimens. Bottom: In situ (Cormorant Island, 125’, 13 Mar 2018) and voucher photos (scale bar is 15 cm) of PSC18 specimen.

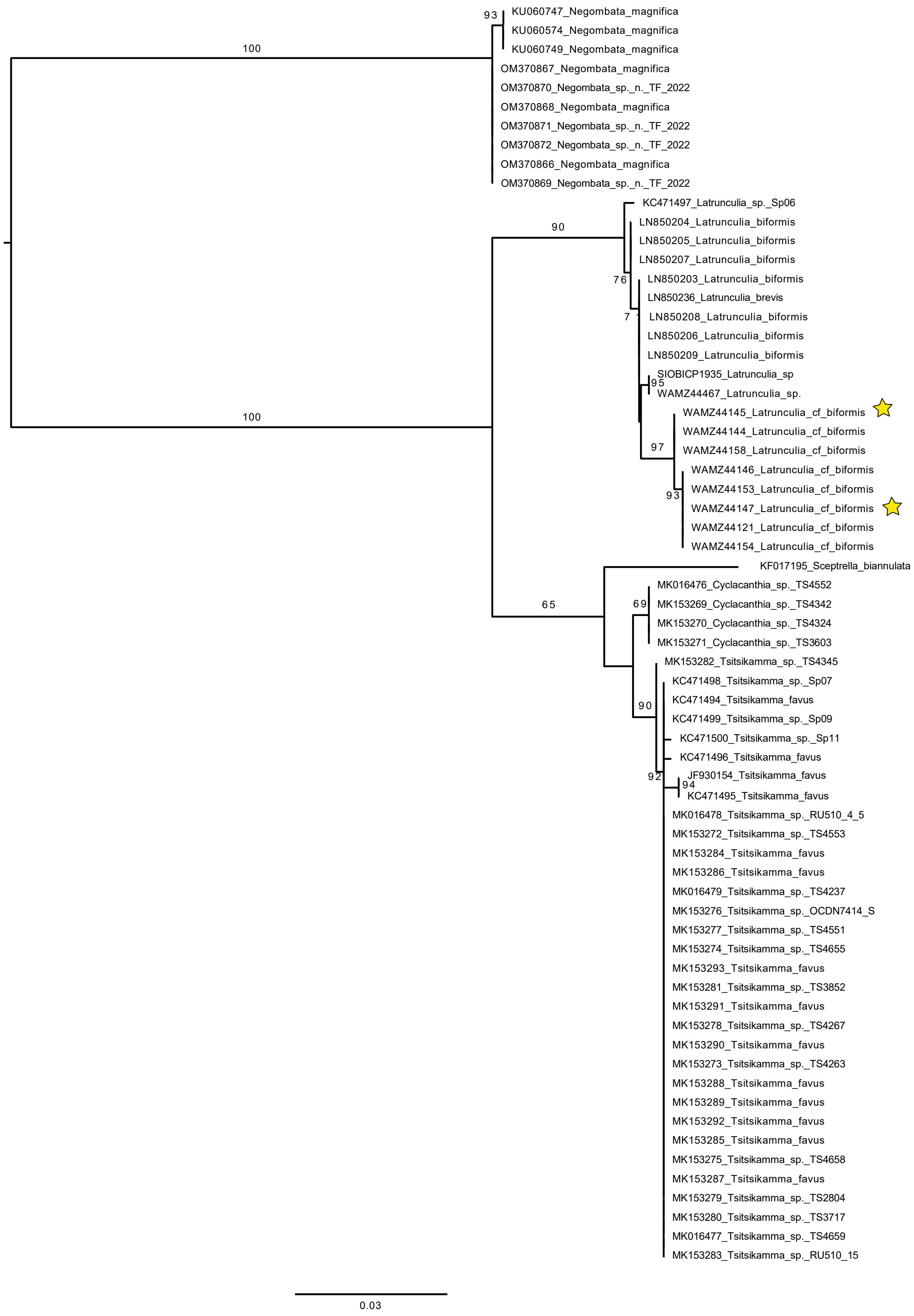

Figure S51. Maximum likelihood tree of Latrunculiidae COI from new specimens and available data on NCBI. Nodes were tested with 1000 ultrafast bootstraps, values below 50 have been removed, as well as short internode values for readability.
